# Potassium Iodide Induces Apoptosis in Salivary Gland Cancer Cells

**DOI:** 10.3390/ijms26115199

**Published:** 2025-05-28

**Authors:** Maksym Skrypnyk, Tetiana Yatsenko, Oleksandra Riabets, Olga Zuieva, Iryna Rodionova, Margarita Skikevych, Yousef Salama, Taro Osada, Morikuni Tobita, Satoshi Takahashi, Nobutaka Hattori, Kazuhisa Takahashi, Koichi Hattori, Beate Heissig

**Affiliations:** 1 Juntendo Biomedical Research Core Facilities, Graduate School of Medicine, Juntendo University, 2-1-1 Hongo, Bunkyo-Ku, Tokyo 113-8421, Japan; maksym.skrypnyk@hdr.mq.edu.au (M.S.); tetyanaa.yatsenko@gmail.com (T.Y.); o.riabets.lb@juntendo.ac.jp (O.R.); o.a.zuieva@gmail.com (O.Z.); irodionova@ukr.net (I.R.); kztakaha@juntendo.ac.jp (K.T.); 2 Centre for Health Informatics, Australian Institute of Health Innovation, Faculty of Medicine, Health and Human Sciences, Macquarie University, 75 Talavera Road, Sydney, NSW 2109, Australia; 3 Department of Enzymes Chemistry and Biochemistry, Palladin Institute of Biochemistry of NAS of Ukraine, 9 Leontovich Street, 01054 Kyiv, Ukraine; 4 Department of Neurosurgery, Graduate School of Medicine, Juntendo University, 2-1-1 Hongo, Bunkyo-Ku, Tokyo 113-8421, Japan; 5 Center for Genome and Regenerative Medicine, Graduate School of Medicine, Juntendo University, 2-1-1 Hongo, Bunkyo-Ku, Tokyo 113-8421, Japan; nhattori@juntendo.ac.jp; 6 Department of Surgical Dentistry and Maxillofacial Surgery with Plastic and Reconstructive Surgery of Head and Neck, Poltava State Medical University, 23 Shevchenko Street, 36011 Poltava, Ukraine; skikevich1959@gmail.com; 7 An-Najah Center for Cancer and Stem Cell Research, Faculty of Medicine and Health Sciences, An-Najah National University, P.O. Box 7, Nablus 99900800, Palestine; yousef.ut@najah.edu; 8 Department of Gastroenterology, Juntendo University Urayasu Hospital, 2-1-1 Tomioka, Urayasu-shi, Chiba 279-0021, Japan; otaro@juntendo.ac.jp; 9 Department of Oral and Maxillofacial Surgery, School of Medicine, Juntendo University, 2-1-1 Hongo, Bunkyo-Ku, Tokyo 113-8421, Japan; mtobita@juntendo.ac.jp; 10 Division of Clinical Precision Research Platform, The Institute of Medical Science, The University of Tokyo, 4 Chome-6-1, Minato-Ku, Tokyo 108-8639, Japan; radius@ims.u-tokyo.ac.jp; 11 Department of Hematology/Oncology, The Institute of Medical Science, The University of Tokyo, 4-6-1, Minato-Ku, Tokyo 108-8639, Japan

**Keywords:** sodium–iodide symporter, iodides, potassium iodide, reactive oxygen species, salivary gland neoplasms, epidermal growth factor, apoptosis, cell proliferation, caspases, BAX, head and neck cancer

## Abstract

Salivary gland cancers (SGCs) pose a therapeutic challenge due to their aggressive nature and limited treatment options. Ion transporters, particularly the sodium/iodide symporter (SLC5A5), which transport iodine in the form of iodide anion (I^−^) into cells, have emerged as potential therapeutic targets in tumors of glandular origin. Our research indicates that SLC5A5 is expressed predominantly in ductal cells of human and murine SGC cells. We assessed the effects of potassium iodide (KI), a source of iodide ions. KI treatment reduced SGC cell proliferation and viability without impacting migration. KI increased ROS levels and triggered caspase-dependent apoptosis, as indicated by the upregulation of the pro-apoptotic protein BAX, downregulation of the anti-apoptotic protein Bcl-2, and induction of SGC cell shrinkage. KI did not affect NF-κB or TNF-α and *SLC5A5* expression. Adding the antioxidant N-acetylcysteine reversed KI-induced growth inhibition, underscoring ROS-induced oxidative stress’s crucial role in growth inhibition. While KI administered in drinking water to mice increased epidermal growth factor (EGF) expression in non-malignant salivary gland tissues, KI decreased EGF receptor (EGFR) expression in malignant SGC cell cultures, where EGFR signaling is frequently dysregulated in SGCs but promoted AKT phosphorylation. Combining KI and anti-EGFR treatment did not yield synergistic anti-SGC cell effects. The study underscores the therapeutic potential of KI as a standalone treatment in vitro for SGC cells. However, the upregulation of EGF in non-malignant tissues and, therefore, the possibility to enhance EGFR-driven signals and AKT phosphorylation after KI treatment in cancer patients could indicate a risk of rendering SGC cells more drug resistant, warranting further investigation to optimize its clinical application.

## 1. Introduction

Salivary gland cancer (SGC) accounts for approximately 0.3% of all newly diagnosed malignancies but is associated with a disproportionately high mortality, contributing to 0.2% of cancer-related deaths [1]. Representing 6–8% of head and neck cancers (HNCs), SGC encompasses various histological types, including mucoepidermoid, adenoid cystic, and acinic cell carcinomas [2,3]. Although surgical resection remains the cornerstone of treatment, anatomical constraints often necessitate a multimodal approach that involves radiotherapy, chemotherapy, and immunotherapy [3,4,5]. However, current therapeutic options are suboptimal: chemotherapy response rates in SGC rarely exceed 45%, and for adenoid cystic carcinoma, they fall below 10%, with considerable systemic toxicity [6,7,8,9,10]. Therefore, identifying specific, molecularly defined targets for more effective and tolerable treatments is a crucial and achievable objective.

The sodium/iodide symporter (SLC5A5, or NIS) is a transmembrane protein responsible for actively taking up iodide through a sodium gradient. In the salivary glands (SG), the expression of SLC5A5 is localized to the basolateral membrane of striated duct cells, enabling iodide concentration in saliva at levels 20–100 times higher than in serum [11,12]. Iodide compounds, such as potassium iodide (KI), have a long clinical history of treating hyperthyroidism, chronic pulmonary disease, and radiation emergencies [13,14,15,16,17]. Both epidemiological and mechanistic evidence support a protective role of iodine against various cancers, including thyroid, breast, and gastric malignancies [13,18,19,20,21,22,23,24]. Despite its recognized anticancer potential, the effects of iodide exposure—specifically high-dose KI—on SGC cell proliferation, apoptosis induction, and oxidative stress remain poorly defined and warrant systematic investigation.

Recent studies suggest that SLC5A5 may exert biological functions beyond iodide transport and could potentially interact with key oncogenic pathways, such as epidermal growth factor (EGF) signaling. Epidermal growth factor receptor (EGFR) is overexpressed in aggressive SGC subtypes and is associated with poor prognosis [25]. As EGF stimulates mitogenic signaling through the PI3K/AKT and MEK/ERK cascades [26,27,28], targeting EGFR has become a clinical strategy in HNC, using agents like cetuximab, trastuzumab, and nimotuzumab [29,30]. Although anti-EGFR therapies demonstrate limited efficacy in SGC, combination strategies may enhance their impact. Preliminary reports have linked iodine and its derivatives to the modulation of EGF-related signaling [31,32]. However, the relationship among SLC5A5 activity, iodide exposure, and EGFR expression or signaling in SG tissues has not been well defined. This study addresses that gap by determining whether excess iodide can modulate EGFR expression and downstream signaling while promoting oxidative stress-induced apoptosis in SGC cells, thus offering a potentially novel therapeutic avenue.

## 2. Results

### 2.1. Enhanced Iodide Uptake in SLC5A5-Expressing SGC Cells

**Figure 1:**
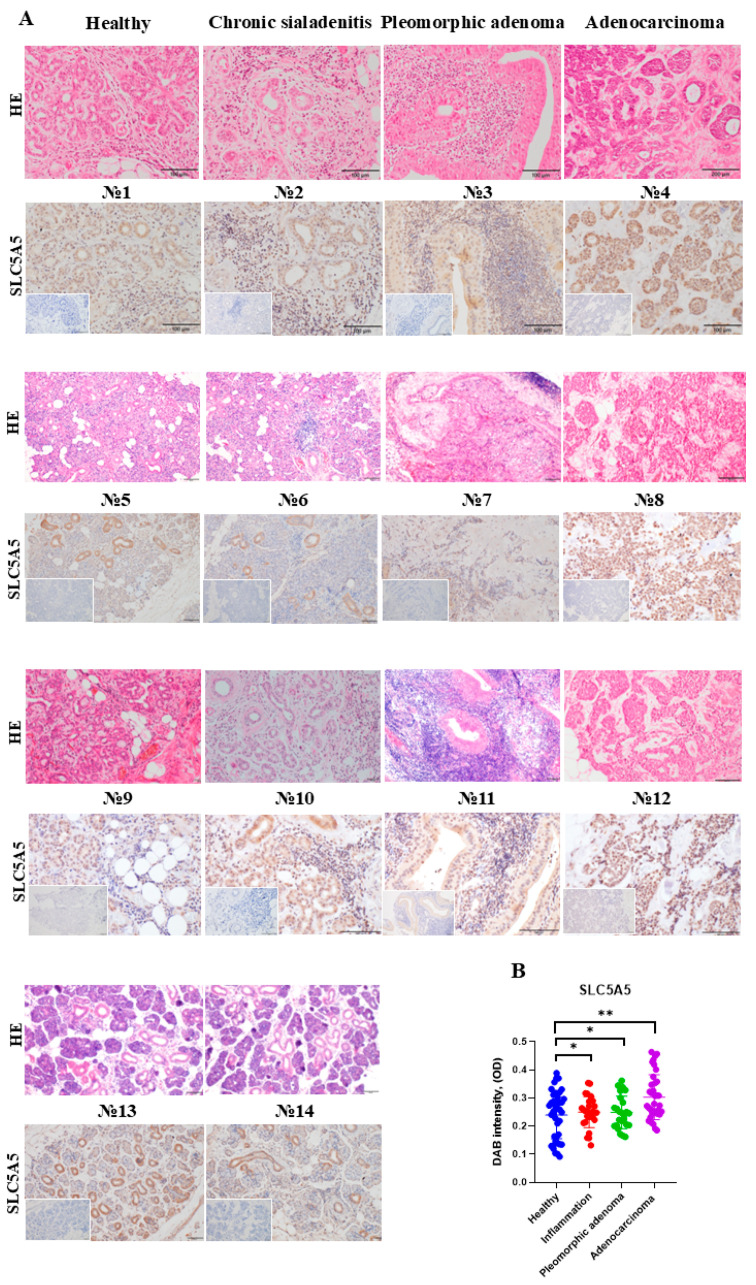
Expression of SLC5A5 by human SG in health and disease. (**A**) Tissue sections of morphologically intact, inflamed (chronic sialadenitis), non-malignant (pleomorphic adenoma), and solid cancer (adenocarcinoma) human SG samples were stained with hematoxylin and eosin (HE; upper images) and for SLC5A5 using immunohistochemistry (brown staining indicates a positive signal; scale bar 200 μm). The isotype control for IHC staining is shown in the lower-left corner of each image; scale bar 200 μm. A description of the clinical samples used is provided in Table 1. (**B**) Quantification of SLC5A5 protein expression, based on IHC staining intensity, measured as the DAB signal per positive structure in examined images (n ≈ 40/group). * *p* < 0.05, ** *p* < 0.01 using a one-way ANOVA test with mean ± standard deviation (SD) depicted.

**Figure 2:**
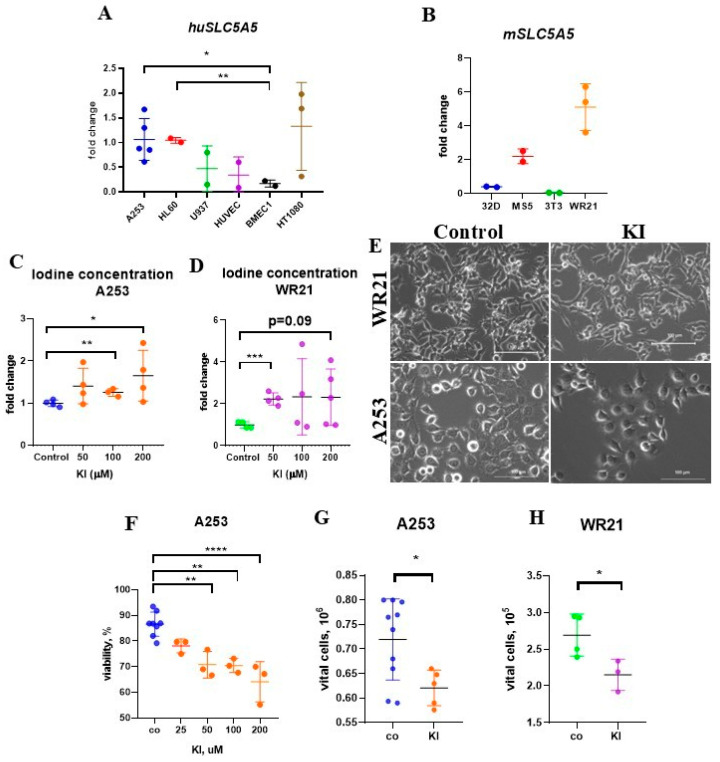
KI reduces the viability of SLC5A5-expressing SGC cells. (**A**,**B**) The fold change in *SLC5A5* expression was determined by qPCR in (**A**) human submandibular SG epidermoid carcinoma (A253; *n* = 5), myeloid hematopoietic (HL-6; *n* = 2, U937; *n* = 2), human, and murine endothelial, respectively (HUVEC; *n* = 2, BMEC1; *n* = 3), as well as in (HT1080; *n* = 3) fibrosarcoma cells and (**B**) murine lymphoblast (32D; *n* = 2) cells, fibroblasts (MS-5; *n* = 2, 3T3; *n* = 2), and submandibular SG adenocarcinoma (WR21; *n* = 3) cells. *SLC5A5* gene expression levels were normalized to *BETA-ACTIN* expression in the same samples, and fold changes were adjusted relative to the expression levels in control samples. (**C**,**D**) The iodine concentration was assessed in cell lysates of A253 (**C**) and WR21 (**D**) cells 48 h after KI treatment at the indicated concentrations (*n* = 4/5 per group). (**E**) Representative light microscopy images of murine WR21 and human A253 SGC cells 48 h after incubation with or without KI (100 μM; scale bar = 100 μm). (**F**) The viability rate of A253 cells treated with the indicated KI concentrations for 48 h was determined by trypan blue exclusion (*n* = 8 for the control (co) group and *n* = 3 for KI 25, 50, 100, and 200 μM groups). (**G**,**H**) The absolute number of viable and control (co) A253 (**G**) and WR21 cells (**H**) after 48 h in culture, following the addition of KI (100 μM), was determined using the trypan blue exclusion assay (*n* = 10 and 5/group for A253 cells and *n* = 4, 3/group for WR21 cells). * *p* < 0.05, ** *p* < 0.01, *** *p* < 0.001, **** *p* < 0.0001 using a one-way ANOVA test (to determine the effects of two independent variables on a dependent variable) or Student’s *t*-test (to compare the performance of two groups under different conditions), with mean ± SD depicted.

We utilized immunohistochemistry to assess SLC5A5 expression in human SG tissues from Caucasian individuals with histologically healthy SG, chronic sialadenitis, pleomorphic adenoma, and adenocarcinoma (Figure 1A). Patients with pre-cancerous conditions or SG adenocarcinoma had not received any treatment before surgery, and their clinical data are presented in Table 1. SLC5A5 expression was observed in ductal cells of SGs, with the highest levels found in ductal cells independent of the underlying disease. The highest expression of SLC5A5 was found in SG tissues of a patient with SG adenocarcinoma (Figure 1B). *SLC5A5* transcripts were detected in the following SG cell lines: the human submandibular SG epidermoid carcinoma (A253) and the mouse submandibular SG adenocarcinoma (WR21), as shown by quantitative PCR (qPCR) (Figure 2A,B). However, non-SGC cell lines also expressed *SLC5A5* to varying extents (Figure 2A,B). At a concentration of 200 μM KI, increased iodine levels were detectable in cell lysates from A253 and WR21 cell lines (Figure 2C,D). These findings indicate that SLC5A5 is expressed in premalignant and malignant human SG tissues. 

### 2.2. KI Treatment Does Not Affect the Migration of SGC Cells

We assessed cell migration in control and KI-treated A253 and WR21 SGC cells using an in vitro scratch assay. KI treatment did not impact cell migration (Figure S1A,B). Furthermore, it did not change the expression levels of metalloproteinases *MMP2* and *MMP9* or the inhibitor *TIMP2* (Figure S1C).

### 2.3. Treatment with KI Impairs the Growth and Viability of SGC Cells

We tested the effects of KI on cell proliferation. Exposure to KI at concentrations above 50 μM decreased the viability of A253 cells and the absolute number of viable cells (Figure 2E–G). Treatment with 100 μM KI for 48 h reduced the percentage of viable cells (data not shown) and the absolute number of viable WR21 cells (Figure 2E,H). These results indicate that KI inhibited cell proliferation and viability in SGC cells.

### 2.4. KI Promotes Apoptosis in SGC Cells

**Figure 3:**
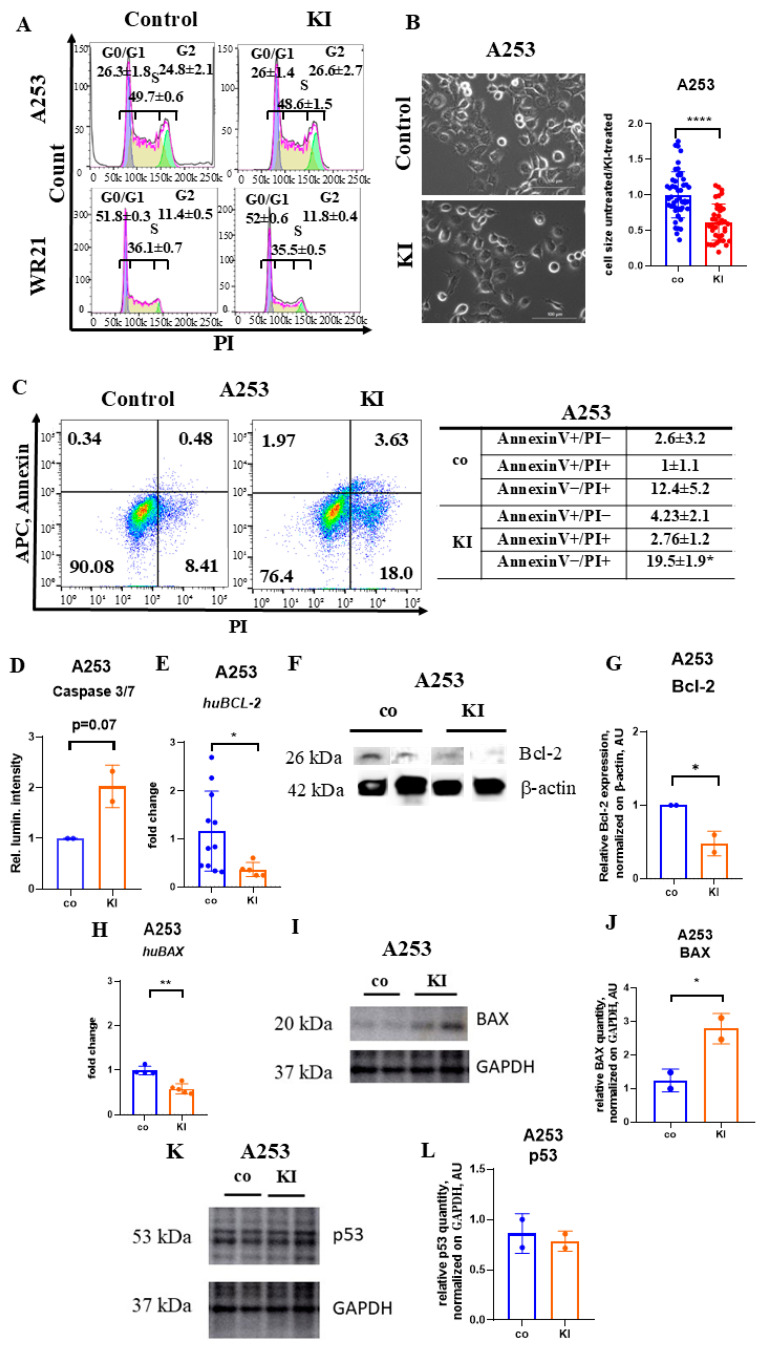
KI treatment induces apoptosis in SGC cells involving the mitochondria-associated molecule BAX. (**A**) A representative histogram illustrates the cell cycle analysis of control and KI-treated A253 and WR21 cells after a 48 h incubation period, assessed by flow cytometry (*n* = 3/group). (**B**) Representative light microscopic images depict human A253 cells treated with KI at 100 μM for 48 h. Scale bar = 100 μm. The panel on the right illustrates the cell size ratio between treated and untreated cells (*n* = 49 and 38 cells/group). (**C**) Flow cytometric analysis evaluates early (AnnexinV+PI-) and late (AnnexinV+PI+) apoptosis in KI-treated or control A253 cells. Means represents three independently conducted experiments. The left panel displays FACS plots of control and KI-treated cells stained with Annexin V-FITC and PI. The right panel presents the statistical analysis of two independent experiments. (**D**) Caspase 3/7 activity in KI-treated and control cells after a 48 h incubation using the Caspase 3/7 activity assay. Signals were normalized to control samples (*n* = 2/group). (**E**) Fold changes in *BCL-2* expression were determined by qPCR in human SGC cell A253 after 48 h of treatment with 100 µM KI. Target gene expression levels were normalized to *BETA-ACTIN* expression in the same samples, and fold changes were adjusted relative to control expression levels (*n* = 11/5 per group). (**F**,**G**) Representative Western blot (**F**) and relative band intensity (**G**) of Bcl-2 and β-actin (internal control) in control and KI-treated (KI 100 µM) A253 cells. Band intensities were calculated by normalizing Bcl-2 expression to β-actin, with comparisons made to the corresponding controls (uncropped Western blot images as Figure S2 (*n* = 2 per group). (**H**) *BAX* expression was determined by qPCR in human SGC cell A253 after 48 h of treatment with 100 µM KI. Target gene expression levels were normalized to *BETA-ACTIN* expression in the same samples, and fold changes were adjusted relative to control expression levels (*n* = 4/5 per group). (**I**,**J**) Representative Western blot (**I**) and relative band intensity (**J**) of BAX and GAPDH (internal control) in control and KI-treated (KI 100 µM) A253 cells. Band intensities were calculated by normalizing BAX expression to GAPDH, with comparisons made to the corresponding controls (uncropped Western blot images as Figure S3B,C (*n* = 2 per group). (**K**,**L**) Representative Western blot (**K**) and relative band intensity (**L**) of p53 and GAPDH (internal control) in control and KI-treated (KI 100 µM) A253 cells. Band intensities were calculated by normalizing p53 quantity to GAPDH (**K**), compared to the corresponding controls (uncropped Western blot images presented as Figure S3A,B; *n* = 2 per group. * *p* < 0.05, ** *p* < 0.05, **** *p* < 0.0001 using a one-way ANOVA test (for determination of the effects of two independent variables on a dependent variable) or Student’s *t*-test (to compare the performance of two groups under different conditions) with mean ± SD depicted.

We subsequently examined the cell cycle using flow cytometry after KI treatment. The KI treatment did not alter the distribution of A253 and WR21 cells across the various phases of the cell cycle (Figure 3A), nor did it change the gene expression levels of the cell cycle regulators *P21*, *P27*, and *CYCLIN D1* (Figure S1D,E), indicating that the impaired cell proliferation following KI treatment was not due to changes in the cell cycle.

Cell shrinkage, or the loss of cell volume, is a common characteristic of programmed cell death that occurs in all instances of apoptosis, regardless of the death stimulus. This reduction in cell volume occurs alongside other classical features of apoptosis [33]. To assess the effect of KI on cell size, A253 cells were exposed to KI for 48 h, and their size was compared to that of untreated controls (Figure 3B). The analysis of light microscopic images revealed that KI-treated cells were smaller than controls, which is compatible with cell shrinkage as a sign of apoptosis (Figure 3B). Flow cytometry data showed an increase in early (Annexin V+PI-) and late (Annexin V+PI+) apoptotic cells in KI-treated samples compared to controls (Figure 3C).

Halogens, such as molecular iodine (I_2_), have been reported to activate programmed cell death through caspase-independent pathways [34]. KI administration activated the effector caspases-3 and -7 in the classical extrinsic and intrinsic apoptosis pathways after 48 h of incubation, but the elevation did not reach statistical significance (*p* = 0.07, *n* = 2) (Figure 3D).

To determine whether KI-mediated apoptosis occurred due to altered expression of the pro-survival factor Bcl-2 and the pro-apoptotic factor BAX—two molecules located in the mitochondrial membrane—qPCR and Western blotting were conducted. A decrease in Bcl-2 transcription and translation was observed 48 h after the addition of KI to A253 cells (Figure 3E–G), along with a reduction in *huBAX* transcription (Figure 3H). In contrast, an increase in BAX protein expression was detected (Figure 3I,J). Cellular stress signals can elevate p53 protein levels. KI did not influence p53 expression in A253 cells (Figure 3K,L). These data suggest that KI promoted caspase-dependent apoptosis, with the upregulation of the pro-apoptotic molecule BAX and the downregulation of the pro-survival protein Bcl-2.

### 2.5. KI and All-Trans Retinoic Acid Treatment Do Not Alter SLC5A5 Expression

Previous studies have shown that combining all-trans retinoic acid (ATRA) and hydrocortisone increases SLC5A5 protein levels and radioactive iodide uptake in MCF-7 human breast cancer cells [35,36]. KI and ATRA treatment did not alter *SLC5A5* transcript levels in WR21 cells (Figure S4A). Similarly, KI did not alter *SLC5A5* expression in A253 cells (Figure S4B).

We evaluated the effects of ATRA and KI as single agents and in combination on WR21 cell proliferation (Figure S4C). ATRA and its carrier DMSO reduced cell proliferation in treated compared to untreated WR21 cells (Figure S4C–E), indicating that the carrier, rather than ATRA, caused the observed growth inhibition. While reconfirming reduced cell viability and lower absolute cell numbers of vital cells in KI-treated cells, no additional growth or viability reduction was found when ATRA was added to KI cultures, and the combined treatment did not differ from KI treatment alone (Figure S4D,E). Neither ATRA alone nor its combination with KI affected *SLC5A5* expression or the growth of SGC cells.

### 2.6. KI Does Not Alter Nuclear Factor Kappa-Light-Chain-Enhancer of Activated B Cells and Tumor Necrosis Factor-Alpha-Expression

**Figure 4:**
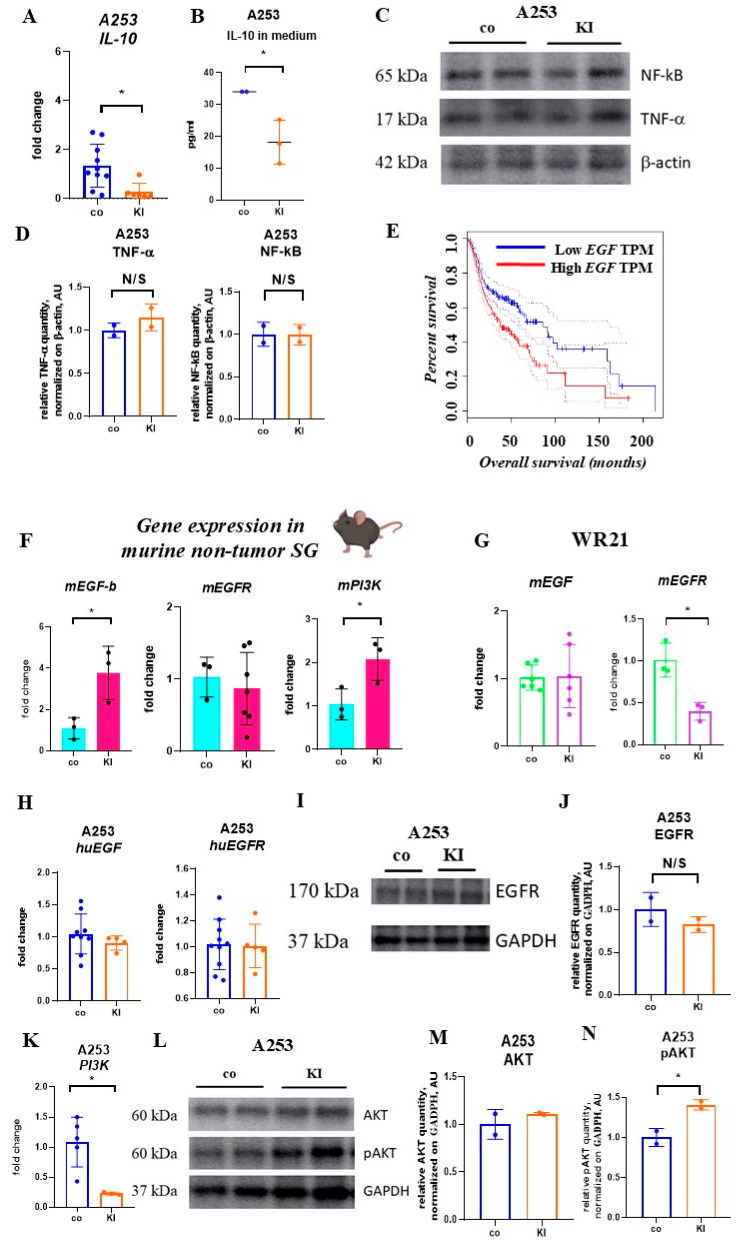
The influence of KI on EGF signaling in cancerous and normal SG cells. (**A**) The fold change in *IL-10* expression levels in human SGC cells A253 after 48 h of treatment with 100 µM KI was measured using qPCR. The target gene expression was normalized to *BETA-ACTIN* expression in the same samples, and fold changes were calculated relative to the control samples (*n* = 10/6 per group). (**B**) IL-10 levels in the culture medium of A253 cells, both treated and untreated, were assessed after 48 h of incubation with 100 µM KI using ELISA (*n* = 3/group). (**C**,**D**) Representative Western blot (**C**) and relative band intensity of TNF-α and NF-κB (**D**), along with β-actin (internal control), in A253 cells that were untreated and treated with 100 µM KI. Band intensities were determined by normalizing the quantity of TNF-α and NF-κB to β-actin compared to the corresponding controls (uncropped Western blot images can be found in Figure S5A–C (*n* = 2/group). (**E**) Kaplan–Meier plots displaying the overall survival of HNSC patients with high (red line) and low (blue) *EGF* mRNA expression (*EGF* Log-rank *p* = 0.00078, HR (high) = 1.6, *p* (HR) = 0.00087, *n* (high) = 257, *n* (low) = 256). Patient stratification based on *EGF* levels was conducted using the GEPIA datasets http://gepia.cancer-pku.cn/ (accessed on 23.12.2024). TPM refers to transcripts per million. (**F**) Fold changes in expression levels of *EGF-b*, *EGFR*, and *PI3K* in submandibular SG tissues from mice drinking regular water (control) or water with KI (1 mg/mL) (*n* = 3/group, except for *EGFR* (KI group), *n* = 7). (**G**,**H**) Fold changes in *mEGF* and *mEGFR* expression levels in WR21 (**G**) and A253 (**H**) cells after 48 h of treatment with 100 µM KI were determined using qPCR. Target gene expression levels were normalized to *BETA-ACTIN* expression in the same samples, and fold changes were calculated relative to the expression levels in control samples (WR21 *mEGF n* = 6/group; *EGFR* = 3/group; A253 *huEGF n* = 9 and 4/group; *huEGFR n* = 10 and 5/group). (**I**,**J**) Representative Western blot (**I**) and relative band intensity of EGFR (**J**) and GAPDH (internal control) in A253 cells, both control and treated with 100 µM KI. Band intensities were calculated by normalizing EGFR quantity to GAPDH compared to the corresponding controls. Uncropped Western blot images are in Figure S3B,D; *n* = 2/group). (**K**) Fold changes in expression levels of *PI3K* in A253 cells after 48 h of treatment with 100 µM KI were determined using qPCR. The target gene expression levels were normalized to *BETA-ACTIN* expression in the same samples, and fold changes were normalized relative to the expression levels in control samples (*n* = 5 and *n* = 3 per group). (**L**–**N**) Representative Western blot (**L**) and the relative band intensity of AKT (**M**), pAKT (**N**), and GAPDH (internal control) in A253 cells, both control and treated with 100 µM KI. Band intensities were calculated by normalizing AKT and pAKT quantity to GAPDH, compared to the respective controls (uncropped Western blot images are shown in (Figure S6A–C). * *p* < 0.05 using Student’s *t*-test (to compare the performance of two groups under different conditions) with mean ± SD depicted.

Recently, our group reported that interleukin 10 (IL-10) enhanced apoptosis in SGC cells by upregulating tumor necrosis factor-alpha (TNF-α) and nuclear factor kappa-light-chain-enhancer of activated B cells (NF-κB) [37]. Although KI addition reduced *IL-10* expression and release into the culture supernatants (Figure 4A,B), it did not alter *NF-κB* or *TNF*-α expression in A253 cells (Figure 4C,D).

### 2.7. KI Enhances EGF Expression in Non-Malignant but Not Malignant SG Cells

High EGFR expression is reported in 74–91% of SGC [38], but a phase II trial indicated insufficient clinical efficacy of cetuximab in treating recurrent, unresectable, or metastatic SGCs [39]. Inorganic iodine regulates EGF production in isolated thyroid cells and controls DNA synthesis and cell proliferation [40]. Since low EGF expression in HNC patients (Figure 4E) is associated with better overall survival, we investigated the effect of KI on the EGFR pathway in both non-malignant and malignant SG tissues. Due to the nature of the cell source, human non-malignant SG cells were unavailable. Therefore, the study on non-malignant SG cells was conducted using mouse SG cells. Consequently, we administered 1 mg/mL of KI in the drinking water to female C57BL/6 mice for 8 weeks. Excess KI in drinking water increased *EGF* but not *EGFR (EGFR1)* expression in murine submandibular SG tissues (Figure 4F). We chose female mice for the murine experiments because it has been reported that SG tumors occur more often in females than in males [3].

In contrast, KI exposure did not affect *EGF* transcript levels; however, it did reduce *EGFR1* expression (here referred to as *EGFR*) in cultured murine WR21 cancer cells (Figure 4G). The addition of KI to cultured human A253 cells (Figure 4H) did not modify the *EGF* transcript levels of *EGFR* and *EGFR2* (*HER2*; data not shown) and did not alter EGFR protein levels (Figure 4I,J). These findings indicate that KI increased *EGF* expression in non-malignant but not malignant SG cells, suggesting that KI can modulate EGFR signaling in non-malignant SG tissues. Phosphatidylinositol 3-kinase (PI3K) is a downstream target of EGFR signaling. Following KI treatment, high *PI3K* expression was observed in non-malignant SG tissues (Figure 4F), while low *PI3K* expression was found in A253 cells (Figure 4K). Cetuximab, an antibody that targets EGFR, did not inhibit A253 cell growth when added at a concentration of 15 µg/mL to cell cultures (data not shown), consistent with reports by others [29]. KI alone suppressed the growth of A253 or WR21 cells, confirming our initial results (data not shown). There was no significant benefit in cetuximab treatment when administered alongside KI (data not shown). Our data suggest that KI suppresses EGFR signaling in SGC cells, but co-treatment with cetuximab did not enhance the anti-tumor response of KI. PI3K/AKT/mTOR signaling is active in over 90% of HNC cases, mainly due to the activation of EGFR (47%) [41]. While KI reduced *PI3K* expression, it did not change AKT (protein kinase B) protein levels but did enhance AKT phosphorylation (Figure 4L–N), a potential downstream target of PI3K.

### 2.8. KI-Driven Reactive Oxygen Species (ROS) Generation Controls SGC Cell Proliferation

**Figure 5:**
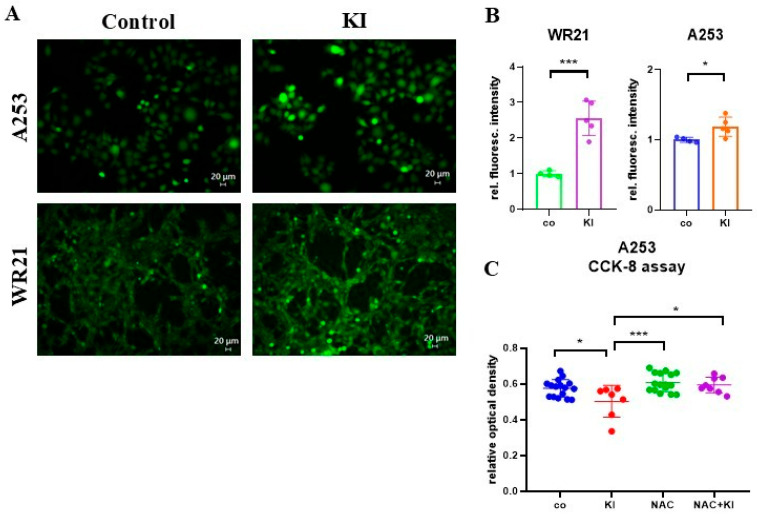
KI induces ROS generation in SGC cells. (**A**) Representative images of fluorescent 2′,7′-dichlorodihydrofluorescein diacetate (DCFH-DA)-stained control and KI-treated A253 and WR21 cells after a 48 h incubation. Bright green fluorescence indicates ROS-producing cells. Scale bar = 20 μm. (**B**) ROS production was assessed by quantifying DCFH-DA fluorescence and normalizing it to total protein in control (*n* = 4/group) and KI-treated (*n* = 5/group) cells. (**C**) The relative optical density values from the CCK-8 assay reflect the viability of A253 cells under various treatment conditions: untreated (*n* = 16/group), treated with KI (*n* = 8/group), treated with N-acetyl-l-cysteine (NAC) (*n* = 16/group), and treated with a combination of KI and NAC (*n* = 8/group). * *p* < 0.05, *** *p* < 0.001 using a one-way ANOVA test (to determine the effects of two independent variables on a dependent variable) or Student’s *t*-test (to compare the performance of two groups under different conditions), with mean ± SD depicted.

Numerous exogenous factors, including environmental pollutants and iodine, can generate ROS [42,43]. Since KI has been shown to induce ROS in mammary cancer cells, and ROS can activate AKT/PKB [44], ROS production was examined in SGC cells. Indeed, KI induced ROS production in human A253 and murine WR21 cells (Figure 5A,B). Significantly, N-acetylcysteine (NAC) was added to the cultures, indicating that the growth inhibition of SGC cells by KI was due to ROS production (Figure 5C).

## 3. Discussion

Ion transporters, including SLC5A5, have been proposed as targets for cancer therapy [45,46]. SLC5A5 is expressed in HNC, but its role in SGC remains poorly understood [12,13,45]. Our research shows that human SGC cells, particularly ductal cells, express SLC5A5. This elevated expression may facilitate greater I_2_ or iodide (I^−^) deposition upon KI administration, potentially increasing the tumor’s sensitivity to treatment. Given its function as an I^−^ transporter, we evaluated the effects of KI on SGC cells in this study.

We found that KI treatment inhibited SGC cell growth and induced caspase-associated apoptosis. Mechanistically, KI upregulated the caspases 3/7, downregulated the pro-survival BCL-2, and upregulated the pro-apoptotic BAX proteins. In thyroid cancer cells, iodine exposure was associated with mitochondrial dysfunction [47]. We found that KI increased ROS production, triggering apoptosis in SGC cells. The addition of the antioxidant NAC reversed KI-induced SGC growth inhibition, possibly through ROS scavenging. By reducing ROS, NAC can counteract the oxidative stress induced by KI [48]. KI and/or iodine induce oxidative stress, leading to apoptosis and growth inhibition in breast, lung cancer, and thyroid cancer cells [34,49,50].

A recent study showed that poly(maleic anhydride-alt-1-octadecene)—coated KI nanoparticles enhance the radiation-induced production of ROS in breast cancer cells [36]. Because our data indicate the uptake of KI in SGC cells, future studies will be needed to test whether KI can sensitize SGC cells to radiotherapy.

I^−^ can induce apoptosis by regulating pro-apoptotic proteins like BAX and BAK via the mitochondrial pathway [34]. We found that p53 levels did not change in KI-treated SGC cells. KI treatment increased BAX protein levels without affecting transcript expression in SGC cells. p53 can induce the transcription of *BAX* in response to DNA damage [51]. One possible explanation for the discrepancy between mRNA and protein BAX levels is that when apoptosis is imminent, protein degradation pathways may be inhibited, resulting in an accumulation of BAX despite decreasing mRNA levels. I_2_ binds to arachidonic acid, which is present at an elevated concentration, and forms 6-iodolactone, activating poly (ADP-ribose) polymerase-1, triggering BAX/caspase-dependent apoptosis [31].

Survival functions are initiated along with the activation of an apoptotic process. AKT, a crucial protein kinase involved in various cellular processes, primarily related to cell growth, survival, and metabolism, is often activated in SGC, promoting tumor growth and survival. Total AKT levels remained unchanged during KI-driven cell growth inhibition, but AKT was activated, as shown by an increase in phosphorylated AKT. Although the mechanism is not examined here, it was reported that low levels of ROS oxidize the disulfide bridges in AKT/PKB, leading to the association of AKT/PKB with PP2A and resulting in the short-term activation of AKT/PKB [52]. It was reported that AKT is activated in response to an apoptotic signal [53]. Given that upregulation of activated AKT often correlates with a more malignant behavior [54], future studies will need to determine whether KI treatment selects for malignant clones that are drug resistant.

KI enhanced the activity of the *NF-κB* transcription factor in thyroid cancer cells [55]. NF-κB activation can downregulate *SLC5A5* expression in thyroid cells, reducing I^−^ uptake [56]. We found that KI did not affect NF-κB and TNF-α expression or change *SLC5A5* expression in SGC cells.

High *EGFR* expression occurs in 74–91% of SGC [29,38]. Antibodies targeting EGFR1/2 have demonstrated tumor-suppressive effects [57], particularly when combined with chemotherapy in HER2-overexpressing tumors [58,59]. Our findings indicate that KI increases *EGF* levels in non-malignant but not in malignant SG cells, as previously reported by Dagogo-Jack [60]. *EGFR* expression did not change after KI treatment. Arroyo-Helguera et al. [31] and others [32] studied the interaction of I_2_ with EGFR signaling. Roesner et al. demonstrated that I_2_ and IL abolished EGF-induced cell growth in SH-SY5Y neuroblastoma and MCF-7 breast cancer cells. This effect was not due to interference with EGF signaling, as I_2_ and IL did not impact the phosphorylation of EGFRs, EGF-induced activation of MAP kinase (Erk 1/2), or EGF-induced lamellar actin protrusion [32].

Non-malignant versus malignant cells showed a different response to KI for *EGF* expression. Increased EGF may promote tissue repair and homeostasis in non-malignant tissues. In vivo, EGF released from KI-treated normal tissues together with KI-mediated unaltered EGFR expression in cancerous cells like SGC cells could drive tumor progression. These differential effects on EGF and EGFR signaling underscore the challenges of KI as a therapeutic target. KI offers selective inhibition of malignant cell growth while preserving normal tissue function.

This study did not directly assess the I^−^ concentration required to control SGC growth in either murine orthotopic models or human patients. However, theoretical estimations suggest that to achieve a systemic I^−^ concentration of 100 µM, an adult weighing 70 kg would need to orally ingest approximately 0.7 g of KI, assuming complete absorption and uniform distribution based on a typical volume of distribution of 0.6 L/kg. It is well established that exocrine glands such as the mammary gland and SGs actively concentrate I^−^, resulting in a 20–50 times higher concentration in breast milk or 10–30 times higher concentration in saliva. Therefore, to achieve a local I^−^ concentration in the SG equivalent to 100 µM in vitro, an oral dose of approximately 6.96–20.85 mg of KI may be sufficient.

Clinically, such doses are feasible. KI is routinely used at doses of 130–250 mg/day for thyroid protection in radiation exposure scenarios or the management of hyperthyroidism, with an established safety profile in adults and adolescents. Accordingly, from a clinical standpoint, the proposed dosing range to achieve therapeutically relevant I^−^ levels in SGs appears plausible. Moreover, supporting evidence from breast cancer studies further strengthens the rationale. For instance, I_2_ at concentrations of 10–40 µM inhibited proliferation of human breast epithelial MCF-12F cells in vitro [31], while in a clinical setting, patients with breast cancer receiving oral I_2_ (5 mg/day) for 7–35 days before surgery demonstrated increased tumor cell apoptosis, reduced side effects, and absence of chemoresistance compared to placebo [61].

These findings collectively support the plausibility of using oral KI to achieve anti-tumor concentrations of I^−^ in salivary tissues. Nevertheless, it remains essential to validate the anticancer effects of KI in well-controlled murine models of SGC and, if successful, proceed with translational studies in human patients. Such investigations will be critical for establishing targeted I^−^ supplementation’s therapeutic relevance and safety in SGC management.

**Figure 6:**
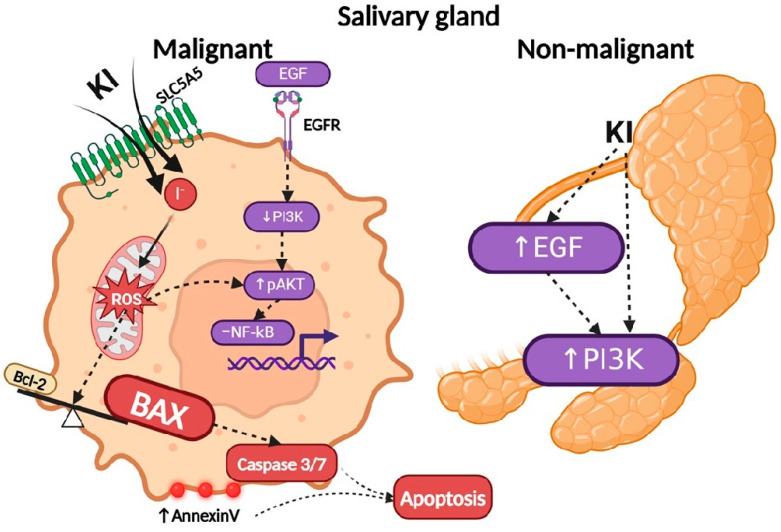
The mechanism of KI action on malignant and non-malignant cells. A schematic illustration depicts the proposed mechanism of KI-driven apoptosis in SGC cells. KI treatment facilitates I^−^ transport into SGC cells via the SLC5A5 transmembrane protein. The SLC5A5-expressing SGC cells can take up iodide (I^−^). KI exposure enhances ROS generation, increases AKT phosphorylation, and activates caspase-dependent apoptosis by upregulating the pro-apoptotic protein BAX and downregulating the anti-apoptotic protein Bcl-2. Unlike in non-malignant SG tissues, KI does not increase EGF expression in SGC cells. Additionally, it downregulates EGF signaling downstream PI3K and does not alter the expression of NF-κB, contrary to its effect in non-malignant tissues. List of abbreviations: pAKT (phosphorylated protein kinase B), BAX (Bcl-2-like protein 4), Bcl-2 (B-cell lymphoma-2), EGFR (epidermal growth factor receptor), EGF (epidermal growth factor), I^−^ (iodide), KI (potassium iodide), PI3K (phosphatidylinositol 3-kinase), ROS (reactive oxygen species), NF-κB (nuclear factor kappa-light-chain-enhancer).

In conclusion, we have demonstrated that malignant and non-malignant pleomorphic adenoma SG tumors express SLC5A5 at higher levels than normal SG tissues, making them potential targets for KI. Our findings show that KI promotes apoptosis via ROS generation, which can be reversed by the ROS antagonist NAC (Figure 6). Normal and malignant SG cells responded differently to KI in their EGF expression.

### Limitation of the Study

This study demonstrated that KI could inhibit the proliferation of SGC cells in vitro by activating apoptosis through the induction of ROS production. This effect was reversed by blocking ROS with NAC. However, it remains unclear whether this is the only KI-activated pathway in SGC cells, necessitating further investigation. Additionally, it is uncertain whether KI administration would have the same effect on tumors in vivo due to the complexity of the tumor microenvironment. Additionally, in vivo experiments are required to address this, such as injecting SGC cells into the SG of animals, followed by treatment with or without KI.

Furthermore, this study considers SGC cells expressing the SLC5A5 symporter exclusively, the primary protein responsible for iodine uptake. Additional research is necessary to determine whether KI’s mechanism of action is mediated through SLC5A5 or another I-transporter. This would involve using an SGC cell line with SLC5A5 deficiency or SLC5A5 overexpression to clarify the role of this symporter in KI-induced effects.

## 4. Materials and Methods

### 4.1. SG Clinical Samples

SG tissue samples were obtained from surgeries performed in the head and neck region on patients with various SG diseases (Institutional Ethics Committee of Poltava State Medical University, protocol numbers 07.04/122 and 236). Before sample collection, all patients provided written informed consent.

### 4.2. HE and SLC5A5 Staining of Human Tissue Sections

Immediately after collection, SG tissues were fixed in 4% paraformaldehyde, and paraffin blocks were generated; 3 μm-thick sections were cut. Tissue sections were stained with HE. The diagnosis was determined based on histopathological findings. Furthermore, tissue sections were blocked and stained with the primary rabbit polyclonal sodium iodine symporter, member 5, (SLC5A5) antibody (Cat. 24324-1-AP, Proteintech, Rosemont, IL, USA) dilution 1:150 overnight at 4 °C followed by staining with secondary antibody goat, anti-rabbit Ig (Cat. E432, Dako, Carpinteria, CA, USA) 1:300. Nuclei were counterstained with hematoxylin (Cat. 4987481295841, Wako, Osaka, Japan). Images were taken using an Olympus BX53 light microscope (Olympus, Tokyo, Japan). The HE images presented in the first row of (Figure 1A) were acquired from separate tissue slides of the same tissue blocks used for other analyses shown elsewhere [37]. Table 1 provides detailed clinical characteristics of the tissue samples used in this study, which were obtained from patients diagnosed with various SG conditions.

A quantification of SLC5A5 staining intensity was conducted using the open-source image analysis software ImageJ 2.9.0 (“Fiji” distribution). Color deconvolution was applied to employ the built-in “H DAB” vector, selecting “Colour_2” to isolate the DAB signal. The resulting image was then converted to 8-bit grayscale. Regions of interest encompassing all DAB-positive structures were manually selected. For each region of interest, the mean gray value was measured. To quantify staining intensity, the mean gray values were converted to optical density (OD) using the following formula: OD = log (Maximum intensity/Mean intensity). For 8-bit images, the maximum intensity was set at 255.

### 4.3. KI and Cytokines

KI (Cat. 7681-11-0, Sigma-Aldrich, Saint Louis, MO, USA) was reconstituted in drinking water (in vivo experiments) and sterile purified Milli-Q water (in vitro experiments), N-acetyl-l-cysteine (NAC; Cat. A7250, Sigma-Aldrich, Saint Louis, MO, USA), anti-EGFR cetuximab (Cat. A2000, Selleck Chemicals, Houston, TX, USA), all-trans retinoic acid (ATRA; Cat. S1653, Selleck Chemicals, USA) was dissolved in dimethyl sulfoxide (DMSO; Cat. 1035920005, Sigma-Aldrich, Saint Louis, MO, USA) at 10 mM, as recommended by the manufacturer and added to cultures at the indicated concentrations.

### 4.4. Cell Lines

The basic media for the human submandibular SG epidermoid carcinoma (A253) cells (Cat. HTB-41, ATCC, Manassas, VA, USA) was McCoy’s 5A (Modified) Medium (Cat. 16600082, Gibco, Grand Island, NY, USA); for U-937 human histiocytic lymphoma cells (Cat. CRL-1593.2, ATCC, Manassas, VA, USA), it was RPMI-1640 medium (Cat. 11875093, Gibco, Grand Island, NY, USA); for HL-60 human promyeloblast cells (Cat. CCL-240, ATCC, Manassas, VA, USA), it was IMDM (Cat. 12440053, Gibco, Grand Island, NY, USA); for HUVEC human umbilical venule endothelial cells (Cat. CRL-1730, ATCC, Manassas, VA, USA), it was F-12K Medium (Cat. 21127022, Gibco, Grand Island, NY, USA) supplemented with heparin (Cat. H3393, Sigma, Saint Louis, MO, USA) and ECGS (Cat. CB-40006, Fisher Scientific, Waltham, MA, USA); for BMEC human bone marrow microvascular endothelial cell (Cat. CRL-3421, ATCC, Manassas, VA, USA), it was MCDB-131 medium (Cat. 10372-019, Gibco, Grand Island, NY, USA); for HT-1080 human fibrosarcoma cells (Cat. CCL-121, ATCC, Manassas, VA, USA), it was MEM (Cat. 137-17215, Wako, Osaka, Japan); for WR21 mouse submandibular SG adenocarcinoma cells (Cat. CRL-2189, ATCC, Manassas, VA, USA) and for murine NIH/3T3 fibroblast (Cat. CRL-1658, ATCC, Manassas, VA, USA), it was D-MEM medium (Cat. 044-29765, FUJIFILM Wako Pure Chemical Corporation, Osaka, Japan); for 32D mouse lymphoblast cells (Cat. CRL-11346, ATCC, Manassas, VA, USA), it was RPMI 1640 (Cat. 11875093, Gibco, Grand Island, NY, USA); and for MS5 murine stromal cells (kindly provided by Dr. MAS Moore, Sloan Kettering Cancer Center, New York, NY, USA), it was IMDM (Cat. 12440053, Gibco, Grand Island, NY, USA). Aside from the basic medium, all media contained 10% FBS (Cat. FB-1380, BioSera, Shiga, Japan) and 1% Penicillin–Streptomycin (P/S; Cat. 15140122, Gibco, Grand Island, NY, USA).

### 4.5. Mice

Eight-week-old female C57BL/6 wild-type mice were purchased from Japan SLC Inc. (Hamamatsu, Japan). The mice were randomly divided into treatment groups: one group received 1 mg/mL KI in their drinking water for 8 weeks, while the control group received plain water without any added drug for the same duration (*n* = 9/group). Animals’ body weight was monitored weekly throughout the experiment, as shown in Figure S4F. After 8 weeks, mice were euthanized, and submandibular SG tissues were extracted for target protein detection and mRNA isolation.

### 4.6. Cell Proliferation or Cytotoxicity Assay After Drug Addition

Cells were cultured in triplicate at 4 × 10^4^/mL for WR21 and 1 × 10^5^/mL for A253 in 6-well plates. Following cell adherence, KI was added to the cultures in concentrations of 25, 50, 100, and 200 μM/mL. If not otherwise indicated, KI was added to cultures at a concentration of 100 μM/mL. After 48 h of incubation, cells were trypsinized with trypsin-EDTA solution (Cat. 205-20255, WAKO, Osaka, Japan). A trypan blue exclusion test was used to determine cell viability. Cells were counted using CellDrop (DeNovix, Wilmington, DE, USA). Images of cultured cells were taken with the All-in-One Fluorescence Microscope, BZ-X series (Keyence, Itasca, IL, USA).

WR21 cells were cultured in triplicate at 4 × 10^4^/mL in 6-well plates. Following cell adherence, KI (100 μM), ATRA (1 μM), DMSO (4 μL), and a combination of ATRA and KI were added to the cultures. After 48 h of incubation, cells were trypsinized and counted, as mentioned above.

### 4.7. Cell Cycle

WR21 and A253 single cells were harvested in PBS plus 2% FBS buffer, washed, and spun at 300× *g* for 5 min. Then, 70% ethanol was added dropwise for cell fixation, and the samples were incubated for 60 min on ice. After two PBS washes, samples were incubated for one hour with RNase A (50 μL of a 100 μg/mL stock solution; Cat. EN0531, Thermo Scientific™, Vilnius, Lithuania). Cells were stained with propidium iodide (PI; 1 μg/mL; Cat. 5135, Tocris Bioscience, Minneapolis, MN, USA) [62]. Stained cells were analyzed using a FACSCelesta™ Cell Analyzer, BD Biosciences (Franklin Lakes, NJ, USA). Cell cycle distribution was analyzed using FlowJo v10 software with the Watson Pragmatic model. Before analysis, doublets were excluded using pulse geometry gating to ensure accurate phase resolution. The univariate algorithm assumes that the DNA content in the G0/G1 and G2/M phases follows a Gaussian distribution. The model initially identifies the G0/G1 peak by locating the channel with the highest cell count on the left side of the DNA content histogram. The standard deviation is estimated at 60% of the peak height, and the fit is refined using least-squares minimization. The G2/M peak is then estimated to be approximately 1.75 times the intensity of the G0/G1 peak and is fitted in a similar manner. The S phase population is calculated as the non-Gaussian area between the G0/G1 and G2/M peaks.

### 4.8. Proliferation CCK-8 Assay

The Cell Counting Kit-8 kit (CCK-8 kit, Cat. CK04, Dojindo, Mashiki, Japan) was used to assess cell viability and proliferation. With the assay, a water-soluble tetrazolium salt (WST-8) reduction into the colored formazan product by cellular dehydrogenases is measured at 450 nm. This assay allows for accurate quantification of metabolically active cells. Specifically, A253 cells (1 × 10^4^/mL) were cultured in 96-well plates. After the cells adhered, KI (100 µM/mL) was added to the cultures, either alone or in combination with NAC (5 µM). A253 and WR21 cells (1 × 10^4^/mL) were cultured in 96-well plates as well. Once the cells adhered, KI (100 µM/mL) was added to the cultures, either alone or with cetuximab (15 µM). The WST-8 reagent (highly water-soluble tetrazolium salt) was introduced 48 h later. Cell dehydrogenase activities reduce the WST-8 reagent, giving yellow-color formazan dye. The generation of formazan dye is directly proportional to the number of living cells. The absorbance of the colored product—formazan—was measured at a wavelength of 450 nm using a microplate reader against 650 nm on the SpectraMax ABS/ABS Plus (Molecular Devices, Silicon Valley, CA, USA), following an incubation period of 1.5 h at 37 °C in 5% CO_2_.

### 4.9. Apoptotic Assay

A253 (5 × 10^4^/mL) were plated in 6-well plates in the presence or absence of KI (100 μM/mL). After a 48 h culture period, cells were trypsinized, washed twice, resuspended with the buffer from the annexin V staining kit, and stained with Annexin V and PI for 20 min (Cat. 556547, BD Pharmingen™ FITC Annexin V Apoptosis Detection Kit I, San Diego, CA, USA). Stained cells were analyzed using the FACSCelesta™ Cell Analyzer BD Biosciences. FlowJo v10 software was used for FACS plot analysis.

### 4.10. Caspase 3/7 Assay

A253 cells were added to 96-chimney well cell microplates (Cat. 655083, Greiner, Frickenhausen, Germany) at a concentration of 1500 cells per well. Cells were cultured for 48 h with/without KI (100 μM/mL) at 37 °C and 5% CO_2_. Culture plates were stored at room temperature for 30 min. Then, the mix of the Caspase-Glo 3/7 substrate and buffer (Cat. G810C, Promega, Madison, WI, USA) was added to the medium at a ratio of 1:1. After 1.5 h at RT, the luminescence was measured with the FlexStation3 Molecular Devices, and the relative luminescence intensity was calculated according to the manufacturer’s protocol.

### 4.11. Detection of Total ROS

Cultured WR21 and A253 cells were stained with 2′,7′-Dichlorodihydrofluorescein diacetate (Cat. D6883, 0000143276, Sigma-Aldrich, St. Louis, MO, USA) [63]. Images of stained cells were taken with the green fluorescent protein channel on an All-in-One Fluorescence Microscope, BZ-X series (Keyence, Itasca, IL, USA). For total ROS quantification, cells were lysed using RIPA Lysis and Extraction Buffer (Cat. 89900, Thermo Scientific, Rockford, IL, USA). After centrifugation, 100 μL of the cell lysate supernatants were transferred into a black 96-well plate. The fluorescence intensity was measured using a fluorescence microplate reader at an excitation wavelength of 485 nm and an emission wavelength of 530 nm with the FlexStation3 (Molecular Devices, Silicon Valley, CA, USA). ROS data were normalized to the total protein concentration in each sample.

### 4.12. Iodine Concentration Detection in Cell Lysate

The concentration of iodine in the cell lysates was determined through colorimetric measurements performed in a microplate using the following reported method [64]. Reagents used include KI, ammonium persulfate (Cat. 7727-54-0, Sigma-Aldrich, Saint Louis, MO, USA), arsenious acid (Cat. 1327-53-3, Sigma-Aldrich, Saint Louis, MO, USA), and ceric ammonium sulfate (Cat. 10378-47-9, Sigma-Aldrich, Saint Louis, MO, USA). The Spectramax 190 Microplate Reader (Molecular Devices, Silicon Valley, CA, USA) was used for measurements. The concentration of iodine in the lysate was normalized to the total protein concentration in the sample. 

### 4.13. Protein Concentration

A colorimetric microplate assay, the DC Protein Assay Kit (Cat. 5000111, Bio-Rad, Hercules, CA, USA), was used to measure protein concentration in cell lysates and tissue samples, serving as a basis for quantitatively measuring ROS activation and iodine concentration detection. The Spectramax 190 Microplate Reader (Molecular Devices, Silicon Valley, CA USA) was used for optical density measurements.

### 4.14. Western Blotting

A253 and WR21 cells were cultured in triplicate at 1 × 10^5^/mL and 4 × 10^4^/mL, respectively, in 6-well plates. KI (100 μM/mL) was added to the cultures following cell adherence. After 48 h of incubation, the medium was removed, and 0.3 mL of RIPA Lysis and Extraction Buffer (Cat. 89900, Thermo Fisher Scientific, Rockford, IL, USA), supplemented with a protease inhibitor cocktail (Cat. 5871, Cell Signaling Technology, Danvers, MA, USA) at a 100:1 ratio, was added. Samples were incubated on ice for 5 min, collected into 1.5 mL Eppendorf tubes, and stored at −80 °C until further use.

The extracted submandibular SGs were placed into tubes containing lysing beads and 0.4 mL of RIPA Lysis and Extraction Buffer (Cat. 89900, Thermo Scientific, Rockford, IL, USA), supplemented with a protease inhibitor cocktail (Cat. 5871, Cell Signaling Technology, Danvers, MA, USA) at a 100:1 ratio. The samples were then homogenized using a Tomy MS-100 Micro Smash homogenizer (Tomy, Tokyo, Japan).

Before usage, protein concentrations were measured in cell lysates using Pierce™ BCA Protein Assay Kits (Cat. 23227, Thermo Fisher Scientific, Waltham, MA, USA), with 70 μg of protein loaded into each well. Before loading, cell lysates were mixed with 6 μL of 4×SDS Sample Buffer and heated for 5 min at 98 °C. Bio-Rad’s tank blotting cells and AE-8135 My Power II 300 (ATTO) equipment were utilized for Western blotting following the manufacturer’s instructions. Precision Plus Protein Dual Color Standards were used at 10 to 250 kDa (Cat. 1610374, BioRad, Hercules, CA, USA). All protein concentrations in each sample were normalized to β-actin or GAPDH (glyceraldehyde 3-phosphate dehydrogenase). Membranes were developed with ECL Prime Western blotting reagent (Cat. RPN2236, Amersham, UK), and images were captured using ImageQuant LAS 4000 (GE Healthcare, Chicago, IL, USA). Quantification of the protein bands was performed with ImageJ software.

Primary AB included mouse anti-β-actin AB (Cat.643801, clone 2F1-1, BioLegend, San Diego, CA, USA) 1:1000; goat polyclonal TNF-alpha (Cat.AF-210-NA, Bio-Techne, Minneapolis, MN, USA) 1.5 µg/mL, rabbit polyclonal anti-NF-κB (Cat. sc-109, Santa Cruz, Dallas, TE, USA) 1:200; rabbit anti-BAX (Cat. 2772, Cell Signaling, Danvers, MA, USA) 1:1000; mouse anti-p53 human monoclonal AB (Cat. sc-98, Santa Cruz, Dallas, TE, USA) 1:1000; mouse anti-GADPH AB (Cat. sc-47724, Santa Cruz, Dallas, TE, USA) 1:200; goat anti-EGFR (Cat. sc-03-G, Santa Cruz, Dallas, TE, USA) 1:200, rabbit anti-AKT (Cat. 9272s, Cell Signaling, Danvers, MA, USA) 1:1000, and rabbit anti-pAKT (Cat. 9271, Cell Signaling, Danvers, MA, USA) 1:2000, mouse monoclonal anti-Bcl-2 AB (C-2) (Cat. sc-7382, Santa Cruz, Dallas, TE, USA) 1:500. The appropriate secondary ABs included goat anti-rabbit IgG-HRP (Cat. sc-2004, Santa Cruz, Dallas, TE, USA) 1:8000, horse anti-mouse HRP-conjugated (Cat. 7076, Cell Signaling, Danvers, MA, USA) 1:2000; and rabbit anti-goat HRP-conjugated (Cat. SA00001- 4, Proteintech, Rosemont, IL, USA) 1:10,000.

### 4.15. Migration Assay

Cultures were initiated with 1 × 10^5^ cells/mL for WR21 and 2 × 10^5^ cells/mL for A253 in standard medium using 12-well plates. When the cells reached confluency, the medium was removed, and the cells were washed once with PBS to eliminate dead cells. A scratch was made across the diameter of the well at the center of the plate using a 100 µL pipette tip, and the scratch borders were marked on the underside of the plate in each well, serving as a landmark for detecting cell migration. After two additional PBS washes, fresh medium with 2% FBS (FBS-reduced) was added to the wells, and cell treatment began. The medium was supplemented with KI (100 μM) and/or metalloproteinase inhibitor—Marimastat 30 μM (Cat. 2631, Tocris Bioscience, Minneapolis, MN, USA)—as a positive control. The All-in-One Fluorescence Microscope, BZ-X series (Keyence, Itasca, IL, USA), was employed to capture an image of the initial scratch size, serving as a baseline for measuring the scratch surface, and again after 24 h of incubation at 37 °C, 5% CO_2_. Images taken immediately after the scratch creation and after 24 h were analyzed using ImageJ software to quantify the cell-free surface area. The percentage of cell migration was calculated using the formula: cell-free surface area after treatment at 24 h × 100% divided by the initial scratch surface area.

### 4.16. ELISA

A 100 µL sample of A253 cell culture medium, collected after 48 h, was used for IL-10 determination with the IL-10 ELISA MAX Deluxe Set (Cat. 431414, BioLegend, San Diego, CA, USA), following the manufacturer’s instructions. A Spectramax 190 Microplate Reader (Molecular Devices, Silicon Valley, CA, USA) was used for optical density measurements.

### 4.17. Cytokine Expression in Cell Lines After Drug Addition

A253 and WR21 cells were cultured in triplicate at 1 × 10^5^/mL and 4 × 10^4^/mL, respectively, in 6-well plates. KI (100 μM) was added to the cultures following cell adherence. After 48 h of incubation, TRIzol LS Reagent (Invitrogen, Carlsbad, CA, USA) was added to adherent cells. After 5 min of incubation on ice, cells were collected and stored at −80 °C for further mRNA extraction.

### 4.18. Quantification of mRNA Expression by qPCR

Total RNA was extracted using RNA TRIzol LS Reagent (Invitrogen, Carlsbad, CA, USA), and cDNA was generated according to the manufacturer’s protocols using a high-capacity cDNA reverse transcription kit (Cat. 4368814, Applied Biosystems, Vilnius, Lithuania). cDNA (10 ng) was used as a template for each PCR amplification using the specific forward and reverse primer pairs in Table 2. For quantitative real-time PCR, PCR mixtures were prepared using TB Green Premix Ex Taq II FAST (Cat. RR830A, Takara, Shiga, Japan) containing 0.2 mM of each primer, and amplification reactions were performed. Gene expression levels were measured using the QuantStudio 3 qPCR System (Applied Biosystems™, Singapore). PCR product levels were estimated by measurement of the intensity of SYBR Green fluorescence. All target gene expressions analyzed in this study were normalized to the housekeeping gene *BETA-ACTIN*.

### 4.19. Bioinformatics Analysis

The RNA sequence data were retrieved from the Gene Expression Profiling Interactive Analysis (GEPIA) database for analysis (http://gepia.cancer-pku.cn/, accessed on 23.12.2024). For Kaplan–Meier (overall survival) curves, we used a median 50 (%) cutoff-high and 50 (%) cutoff-low and a 95% confidence interval [65].

### 4.20. Statistical Analysis

GraphPad PRISM 8.0.1 by Dotmatics was used for statistical data processing. All quantitative data are presented as mean ± SD, as this measure better represents the variability and reliability of the group mean in biological experiments. *p*-values < 0.05 were considered statistically significant. Data distributions were tested for normality using the Shapiro–Wilk normality test. Outliers were checked using the ROUT test and removed (α = 0.05). For data analysis, we used a one-way ANOVA test (to determine the effects of two independent variables on a dependent variable) or a student’s *t*-test (to compare the performance of two groups under different conditions). The difference between groups was considered statistically significant at *p* < 0.05.

### 4.21. Cultured Cell Image Analysis

Analysis of the photos of cultured cells was conducted with ImageJ software (Rasband, W.S., ImageJ, U. S. National Institutes of Health, Bethesda, MD, USA). The freehand selection tool was used to outline and measure the surface area of each cell individually.

## Figures and Tables

**Table 1:** Patient characteristics of the clinical samples of SG used in the study.

Sample Number	Diagnosis, Histological Findings	Treatment	Age	Gender
1	Calculous sialadenitis of the right submandibular SG. Pathological biopsy included adjacent tissues; morphologically healthy tissue in the biopsy material.	The entire submandibular SG was extracted.	48	Male
2	Chronic sialadenitis caused by multiple concrements in the parenchyma of the submandibular SGs. Chronic inflammatory changes in glandular tissue.	The entire glands were extracted.	60	Female
3	Pleomorphic adenoma of the right parotid SG. Benign mixed tumor characteristics in the glandular tissue.	The tumor was surgically excised along with the adjacent parotid SG.	34	Female
4	Adenoid cystic carcinoma of the left parotid SG. Malignant tumor with perineural invasion and a characteristic cribriform pattern (Tissue block 4).	The tumor, along with the entire SG, was surgically excised.	45	Female
5	Calculous sialadenitis of the right submandibular SG. Morphologically healthy SG tissue in the biopsy material.	The entire SG was surgically excised.	40	Male
6	Calculous sialadenitis of the right submandibular SG. Inflammatory changes in the tissue biopsy material.	The entire SG was surgically excised.	40	Male
7	Pleomorphic adenoma of the left parotid SG. Benign mixed tumor characteristic of pleomorphic adenoma.	The tumor was surgically excised along with the adjacent parotid SG.	82	Female
8	Adenoid cystic carcinoma of the left parotid SG. Malignant tumor with perineural invasion and a characteristic cribriform pattern (Tissue block 5).	The entire SG was surgically extracted.	45	Female
9	Calculous sialadenitis of the left submandibular SG. Morphologically intact and healthy biopsy material.	The entire SG was surgically extracted.	31	Male
10	Exacerbation of chronic submandibular sialadenitis. Inflammatory changes in the submandibular SG.	The entire SG was surgically extracted.	42	Male
11	Pleomorphic adenoma of the right submandibular SG. Presence of a benign pleomorphic adenoma.	The tumor was surgically excised along with the adjacent parotid SG.	51	Male
12	Adenoid cystic carcinoma of the left parotid SG. Malignant tumor with perineural invasion and a characteristic cribriform pattern. The entire gland was surgically excised (Tissue block 2).	The tumor, along with the entire SG, was surgically excised.	45	Female
13	Calculous sialadenitis of the left submandibular SG. Morphologically healthy tissue in the biopsy material.	Extirpation of the affected SG.	46	Female
14	Calculous sialadenitis of the left submandibular SG. Inflammatory changes consistent with chronic sialadenitis.	Extirpation of the affected SG.	46	Female

**Table 2:** Primers used for RT-qPCR.

Target	Forward	Reverse
	Mouse	
*BETA-ACTIN*	GGCTGTATTCCCCTCCATCG	CCAGTTTGGTAACAATGCCATGT
*PI3K*	CCTGTAGCTGTTTACAATGAGA	GGCAATCTAATGAGCTTCTTCA
*P21*	TCGCTGTCTTGCACTCTGGTGT	CCAATCTGCGCTTGGAGTGATAG
*P27*	AGCAGTGTCCAGGGATGAGGAA	TTCTTGGGCGTCTGCTCCACAG
*SLC5A5*	GCTAATTCGCTGCTCACAG	GCTGAAGCGCAGTTCTAGGT
*CYCLIND1*	GCAGAAGGAGATTGTGCCATCC	AGGAAGCGGTCCAGGTAGTTCA
*EGF*	ACTGGTGTGACACCAAGAGGTC	CCACAGGTGATCCTCAAACACG
*EGFR*	GGACTGTGTCTCCTGCCAGAAT	GGCAGACATTCTGGATGGCACT
*MMP9*	AGACGACATAGACGGCATCC	TCGGCTGTGGTTCAGTTGT
*MMP2*	GGCAGTGCAATACCTGAACA	GCCCAAAGAACTTCTGCATC
*TIMP2*	AGCCAAAGCAGTGAGCGAGAAG	GCCGTGTAGATAAACTCGATGTC
	Human	
*SLC5A5*	CCAGGGAGAGGGACAGACAG	GGCTCCCTGGGTTAGGAATC
*P21*	AGGTGGACCTGGAGACTCTCAG	TCCTCTTGGAGAAGATCAGCCG
*P27*	CTGCAACCGACGATTCTTCT	GCATTTGGGGAACCGTCTGA
*CYCLIND1*	CCGTCCATGCGGAAGATC	GAAGACCTCCTCCTCGCACT
*BAX*	CATGGAGCTGCAGAGGATGA	CCAGTTGAAGTTGCCGTCAG
*BCL-2*	GGATAACGGAGGCTGGGATG	GGGCCAAACTGAGCAGAGTC
*IL-10*	GGCACCCAGTCTGAGAACAG	ACTCTGCTGAAGGCATCTCG
*BETA-ACTIN*	CCAACCGCGAGAAGATGA	CCAGAGGCGTACAGGGATAG
*EGF*	ACGCCCTAAGTCGAGACCGGA	TGGCTGCATTCAGACACATTGCG
*EGFR*	AACACCCTGGTCTGGAAGTACG	TCGTTGGACAGCCTTCAAGACC
*PI3K*	GAAGCACCTGAATAGGCAAGTCG	GAGCATCCATGAAATCTGGTCGC

## Data Availability

All data relevant to this research are included in the publication.

## Supplementary Materials

The following supporting information can be downloaded at: https://www.mdpi.com/article/10.3390/ijms26115199/s1.

## Abbreviations

AKT (protein kinase B), ATRA (all-trans retinoic acid), BAX (Bcl-2-like protein 4), Bcl-2 (B-cell lymphoma-2), DCFH-DA (2′,7′-dichlorodihydrofluorescein diacetate), EGFR (epidermal growth factor receptor), EGF (epidermal growth factor), GAPDH (glyceraldehyde 3-phosphate dehydrogenase), HE (hematoxylin and eosin), HER2 (EGF receptor 2), HNCs (head and neck cancers), I_2_ (molecular iodine), I^−^ (iodide), IL (iodolactone), IL-10 (interleukin 10), KI (potassium iodide), NAC (N-acetyl-l-cysteine), NIS (sodium/iodide symporter), PI3K (phosphatidylinositol 3-kinase), qPCR (quantitative PCR), ROS (reactive oxygen species), SG (salivary gland), SGC (salivary gland cancer), TNF-α (tumor necrosis factor-alpha), NF-κB (nuclear factor kappa-light-chain-enhancer).

## References

[1] Sung H., Ferlay J., Siegel R.L., Laversanne M., Soerjomataram I., Jemal A., Bray F. (2021). Global cancer Statistics 2020: GLOBOCAN estimates of incidence and mortality worldwide for 36 cancers in 185 countries. CA Cancer J. Clin..

[2] Carvalho A.L., Nishimoto I.N., Califano J.A., Kowalski L.P. (2005). Trends in incidence and prognosis for head and neck cancer in the United States: A Site-specific Analysis of the SEER Database. Int. J. Cancer.

[3] Steuer C.E., Hanna G.J., Viswanathan K., Bates J.E., Kaka A.S., Schmitt N.C., Ho A.L., Saba N.F. (2023). The evolving landscape of salivary gland tumors. CA Cancer J. Clin..

[4] Geiger J.L., Ismaila N., Beadle B., Caudell J.J., Chau N., Deschler D., Glastonbury C., Kaufman M., Lamarre E., Lau H.Y. (2021). Management of salivary gland malignancy: ASCO Guideline. J. Clin. Oncol..

[5] Zhang Q., Yang Y., Yang P., Tan Y., Liu X., Xiong B., Qiu J. (2020). Cosmetic approach selection in parotidectomy for benign parotid gland tumour according to its location. J. Plast. Reconstr. Aesthetic Surg..

[6] Airoldi M., Pedani F., Succo G., Gabriele A.M., Ragona R., Marchionatti S., Bumma C. (2001). Phase II randomized trial comparing vinorelbine versus vinorelbine plus cisplatin in patients with recurrent salivary gland malignancies. Cancer.

[7] Nakano K., Sato Y., Sasaki T., Shimbashi W., Fukushima H., Yonekawa H., Mitani H., Kawabata K., Takahashi S. (2016). Combination chemotherapy of carboplatin and paclitaxel for advanced/metastatic salivary gland carcinoma patients: Differences in responses by different pathological diagnoses. Acta Otolaryngol..

[8] Laurie S.A., Siu L.L., Winquist E., Maksymiuk A., Harnett E.L., Walsh W., Tu D., Parulekar W.R. (2010). A Phase 2 study of platinum and gemcitabine in patients with advanced salivary gland cancer. Cancer.

[9] Locati L.D., Cavalieri S., Bergamini C., Resteghini C., Alfieri S., Calareso G., Bossi P., Perrone F., Tamborini E., Quattrone P. (2019). Phase II trial with axitinib in recurrent and/or metastatic salivary gland cancers of the upper aerodigestive tract. Head Neck.

[10] Tchekmedyian V., Sherman E.J., Dunn L., Tran C., Baxi S., Katabi N., Antonescu C.R., Ostrovnaya I., Haque S.S., Pfister D.G. (2019). Phase II study of lenvatinib in patients with progressive, recurrent or metastatic adenoid cystic carcinoma. J. Clin. Oncol..

[11] La Perle K.M.D., Kim D.C., Hall N.C., Bobbey A., Shen D.H., Nagy R.S., Wakely P.E., Lehman A., Jarjoura D., Jhiang S.M. (2013). Modulation of sodium/iodide symporter expression in the salivary gland. Thyroid.

[12] Mandel S.J., Mandel L. (2003). Radioactive iodine and the salivary glands. Thyroid.

[13] Aceves C., Anguiano B., Delgado G. (2013). The extrathyronine actions of iodine as antioxidant, apoptotic, and differentiation factor in various tissues. Thyroid.

[14] Oriel J.D. (1994). The Scars of Venus.

[15] Brouse S.D., Johnson M.L., Muldrew-Jones K.M. (2009). Iodine-containing compounds. Comprehensive Handbook of Iodine.

[16] Xue S., Gu R., Wu T., Zhang M., Wang X., Wu T. (2006). Oral potassium iodide for the treatment of sporotrichosis. Cochrane Database of Systematic Reviews.

[17] Thotan S.P., Kumar V., Gupta A., Mallya A., Rao S. (2010). Subcutaneous phycomycosis—Fungal infection mimicking a soft tissue tumor: A case report and review of literature. J. Trop. Pediatr..

[18] Sakafu L.L., Mselle T.F., Mwaiselage J.D., Maunda K.K., Eddin B.S., Zafereo M.E. (2018). Thyroid cancer and iodine deficiency status: A 10-year review at a single cancer center in Tanzania. OTO Open.

[19] Zimmermann M.B., Galetti V. (2015). Iodine intake as a risk factor for thyroid cancer: A comprehensive review of animal and human studies. Thyroid Res..

[20] Manjer J., Sandsveden M., Borgquist S. (2020). Serum iodine and breast cancer risk: A prospective nested case–control study stratified for selenium levels. Cancer Epidemiol. Biomark. Prev..

[21] Gulaboglu M., Yildiz L., Celebi F., Gul M., Peker K. (2005). Comparison of iodine contents in gastric cancer and surrounding normal tissues. Clin. Chem. Lab. Med. CCLM.

[22] Kwon Y.-J., Lee H.-S., Kang S.-W., Lee J.-W. (2024). Association between consumption of iodine-rich foods and thyroid cancer prevalence: Findings from a large population-based study. Nutrients.

[23] Stadel B. (1976). Dietary iodine and risk of breast, endometrial, and ovarian cancer. Lancet.

[24] Cann S.A., van Netten J.P., van Netten C. (2000). Hypothesis: Iodine, selenium and the development of breast cancer. Cancer Causes Control.

[25] Zimmermann M., Zouhair A., Azria D., Ozsahin M. (2006). The epidermal growth factor receptor (egfr) in head and neck cancer: Its role and treatment implications. Radiat. Oncol..

[26] Franke T.F., Kaplan D.R., Cantley L.C., Toker A. (1997). Direct regulation of the Akt proto-oncogene product by phosphatidylinositol-3,4-bisphosphate. Science.

[27] Ren Y., Hong Y., He W., Liu Y., Chen W., Wen S., Sun M. (2022). EGF/EGFR promotes salivary adenoid cystic carcinoma cell malignant neural invasion via activation of PI3K/AKT and MEK/ERK signaling. Curr. Cancer Drug Targets.

[28] Ni W., Chen Z., Zhou X., Yang R., Yu M., Lu J., Kaye F.J., Wu L. (2021). Targeting Notch and EGFR signaling in human mucoepidermoid carcinoma. Signal Transduct. Target. Ther..

[29] Kuroda M., Komatsu N., Kosai A., Hamakubo T., Abe T. (2024). Anti-EGFR antibody immunotoxins improve cytotoxic effects in the salivary gland cancer A253 cell Line. J. Oral Maxillofac. Surg. Med. Pathol..

[30] Huang Y., Yu T., Fu X., Chen J., Liu Y., Li C., Xia Y., Zhang Z., Li L. (2013). EGFR inhibition prevents in vitro tumor growth of salivary adenoid cystic carcinoma. BMC Cell Biol..

[31] Arroyo-Helguera O., Rojas E., Delgado G., Aceves C. (2008). Signaling pathways involved in the antiproliferative effect of molecular iodine in normal and tumoral breast cells: Evidence that 6-iodolactone mediates apoptotic effects. Endocr. Relat. Cancer.

[32] Rösner H., Torremante P., Möller W., Gärtner R. (2009). Antiproliferative/cytotoxic activity of molecular iodine and iodolactones in various human carcinoma cell lines. no interfering with EGF-signaling, but evidence for apoptosis. Exp. Clin. Endocrinol. Diabetes.

[33] Bortner C.D., Cidlowski J.A. (2003). Uncoupling cell shrinkage from apoptosis reveals that Na+ influx is required for volume loss during programmed cell death. J. Biol. Chem..

[34] Shrivastava A., Tiwari M., Sinha R.A., Kumar A., Balapure A.K., Bajpai V.K., Sharma R., Mitra K., Tandon A., Godbole M.M. (2006). Molecular iodine induces caspase-independent apoptosis in human breast carcinoma cells involving the mitochondria-mediated pathway. J. Biol. Chem..

[35] Dohán O., De la Vieja A., Carrasco N. (2006). Hydrocortisone and purinergic signaling stimulate sodium/iodide symporter (NIS)-mediated iodide transport in breast cancer cells. Mol. Endocrinol..

[36] Cline B.L., Jiang W., Lee C., Cao Z., Yang X., Zhan S., Chong H., Zhang T., Han Z., Wu X. (2021). Potassium iodide nanoparticles enhance radiotherapy against breast cancer by exploiting the sodium-iodide symporter. ACS Nano.

[37] Skrypnyk M., Yatsenko T., Riabets O., Salama Y., Skikevych M., Osada T., Tobita M., Takahashi S., Hattori K., Heissig B. (2024). Interleukin-10 induces TNF-driven apoptosis and ROS production in salivary gland cancer cells. Heliyon.

[38] Guazzo E., Cooper C., Wilkinson L., Feng S., King B., Simpson F., Porceddu S., Panizza B., Coward J.I.G. (2021). Therapeutic implications of immune-profiling and EGFR expression in salivary gland carcinoma. Head Neck.

[39] Locati L.D., Bossi P., Perrone F., Potepan P., Crippa F., Mariani L., Casieri P., Orsenigo M., Losa M., Bergamini C. (2009). Cetuximab in recurrent and/or metastatic salivary gland carcinomas: A phase II study. Oral Oncol..

[40] Tramontano D., Veneziani B.M., Lombardi A., Villone G., Ingbar S.H. (1989). Iodine inhibits the proliferation of rat thyroid cells in culture. Endocrinology.

[41] Marquard F.E., Jücker M. (2020). PI3K/AKT/MTOR signaling as a molecular target in head and neck cancer. Biochem. Pharmacol..

[42] Arbez-Evangelista C., Arroyo-Xochihua O., Ortega-Ibarra I.H., Ortega-Ibarra E., De León-Ramírez Y.M., Cuevas-Romero E., Arroyo-Helguera O. (2024). Excess iodine consumption induces oxidative stress and pancreatic damage independently of chemical form in male wistar rats: Participation of PPAR-γ and C/EBP-β. Biology.

[43] De Almeida A.J.P.O., de Oliveira J.C.P.L., da Silva Pontes L.V., de Souza Júnior J.F., Gonçalves T.A.F., Dantas S.H., de Almeida Feitosa M.S., Silva A.O., de Medeiros I.A. (2022). ROS: Basic concepts, sources, cellular signaling, and its implications in aging pathways. Oxidative Med. Cell. Longev..

[44] Murata H., Ihara Y., Nakamura H., Yodoi J., Sumikawa K., Kondo T. (2003). Glutaredoxin Exerts an antiapoptotic effect by regulating the redox state of Akt. J. Biol. Chem..

[45] Riesco-Eizaguirre G., Santisteban P., De la Vieja A. (2021). The complex regulation of NIS expression and activity in thyroid and extrathyroidal tissues. Endocr. Relat. Cancer.

[46] Oh J.M., Ahn B.-C. (2021). Molecular mechanisms of radioactive iodine refractoriness in differentiated thyroid cancer: Impaired sodium iodide symporter (NIS) expression owing to altered signaling pathway activity and intracellular localization of NIS. Theranostics.

[47] Upadhyay G., Singh R., Sharma R., Balapure A.K., Godbole M.M. (2002). Differential action of iodine on mitochondria from human tumoral- and extra-tumoral tissue in inducing the release of apoptogenic proteins. Mitochondrion.

[48] Sun S.-Y. (2010). N-Acetylcysteine, reactive oxygen species and beyond. Cancer Biol. Ther..

[49] Zhang L., Sharma S., Zhu L.X., Kogai T., Hershman J.M., Brent G.A., Dubinett S.M., Huang M. (2003). Nonradioactive iodide effectively induces apoptosis in genetically modified lung cancer cells. Cancer Res..

[50] Vitale M., Di Matola T., D’Ascoli F., Salzano S., Bogazzi F., Fenzi G., Martino E., Rossi G. (2000). Iodide excess induces apoptosis in thyroid cells through a p53-independent mechanism involving oxidative stress. Endocrinology.

[51] Toshiyuki M., Reed J.C. (1995). Tumor suppressor p53 is a direct transcriptional activator of the human Bax gene. Cell.

[52] Zhang J., Wang X., Vikash V., Ye Q., Wu D., Liu Y., Dong W. (2016). ROS and ROS-mediated cellular signaling. Oxidative Med. Cell. Longev..

[53] Tang D., Okada H., Ruland J., Liu L., Stambolic V., Mak T.W., Ingram A.J. (2001). Akt is activated in response to an apoptotic signal. J. Biol. Chem..

[54] Nakatani K., Thompson D.A., Barthel A., Sakaue H., Liu W., Weigel R.J., Roth R.A. (1999). Up-regulation of Akt3 in estrogen receptor-deficient breast cancers and androgen-independent prostate cancer lines. J. Biol. Chem..

[55] Liu M., Huang J., Wang J., Hu S., Li S., Xiong C., Liu F., Yuan C., Hu Y., Sun W. (2021). Potassium iodide promotes the pyroptosis of thyroid follicular epithelial cells through the PARP1-NF-ΚB-NLRP3 inflammasome. Chin. J. Endocrinol. Metab..

[56] Faria M., Domingues R., Paixão F., Bugalho M.J., Matos P., Silva A.L. (2020). TNFα-mediated activation of NF-ΚB downregulates sodium-iodide symporter expression in thyroid cells. PLoS ONE.

[57] Haddad R., Colevas A.D., Krane J.F., Cooper D., Glisson B., Amrein P.C., Weeks L., Costello R., Posner M. (2003). Herceptin in patients with advanced or metastatic salivary gland carcinomas. A phase II study. Oral Oncol..

[58] Takahashi H., Tada Y., Saotome T., Akazawa K., Ojiri H., Fushimi C., Masubuchi T., Matsuki T., Tani K., Osamura R.Y. (2019). Phase II trial of trastuzumab and docetaxel in patients with human epidermal growth factor receptor 2–positive salivary duct carcinoma. J. Clin. Oncol..

[59] Kurzrock R., Bowles D.W., Kang H., Meric-Bernstam F., Hainsworth J., Spigel D.R., Bose R., Burris H., Sweeney C.J., Beattie M.S. (2020). Targeted therapy for advanced salivary gland carcinoma based on molecular profiling: Results from MyPathway, a phase iia multiple basket study. Ann. Oncol..

[60] Dagogo-jack S. (1994). Dietary iodine affects epidermal growth factor levels in mouse thyroid and submaxillary glands. Endocr. Res..

[61] Moreno-Vega A., Vega-Riveroll L., Ayala T., Peralta G., Torres-Martel J.M., Rojas J., Mondragón P., Domínguez A., De Obaldía R., Avecilla-Guerrero C. (2019). Adjuvant Effect of Molecular Iodine in Conventional Chemo-therapy for Breast Cancer. Randomized Pilot Study. Nutrients.

[62] Crissman H.A., Steinkamp J.A. (1973). Rapid, simultaneous measurement of DNA, protein, and cell volume in single cells from large mammalian cell populations. J. Cell Biol..

[63] Kim H., Xue X. (2020). Detection of total reactive oxygen species in adherent cells by 2’,7’-dichlorodihydrofluorescein diacetate staining. J. Vis. Exp..

[64] Ohashi T., Yamaki M., Pandav C.S., Karmarkar M.G., Irie M. (2000). Simple microplate method for determination of urinary iodine. Clin. Chem..

[65] Tang Z., Li C., Kang B., Gao G., Li C., Zhang Z. (2017). GEPIA: A web server for cancer and normal gene expression profiling and interactive analyses. Nucleic Acids Res..

