# Supplementary material for: Potassium Iodide Induces Apoptosis in Salivary Gland Cancer Cells
Source: Int J Mol Sci. 2025 May 28;26(11):5199. doi: 10.3390/ijms26115199 (PMC12155144; doi:10.3390/ijms26115199)
Supplement: Supplementary file 1 [file ijms-26-05199-s001.zip › ijms-3451411-supplementary.pdf]

Supplementary Figures S1-S6

Article Title: Potassium Iodide Induces Apoptosis in Salivary Gland Cancer Cells

Maksym Skrypnyk, Tetiana Yatsenko, Oleksandra Riabets, Olga Zuieva, Iryna Rodionova, Margarita Skikevych, Yousef Salama, Taro Osada, Morikuni Tobita, Satoshi Takahashi, Nobutaka Hattori, Kazuhisa Takahashi, Koichi Hattori and Beate Heissig

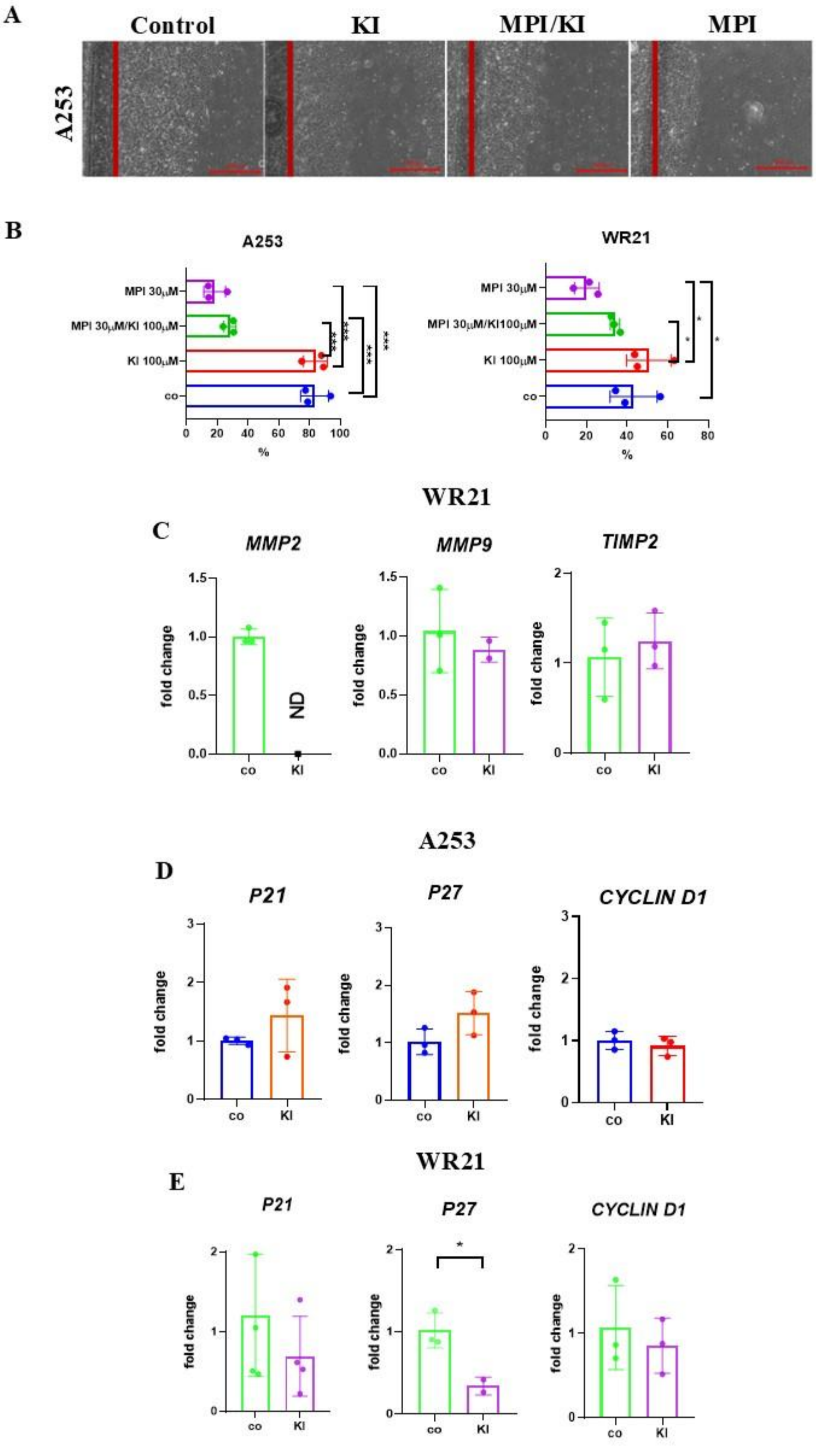

Supplementary Figure S1. KI treatment does not affect the migration of SGC cells or alter the cell cycle. A. Representative images of the migration assay for A253 cells treated with or without KI after a scratch was made on confluent cell layers. Migration was assessed by counting the cells that migrated into the scraped area 24 hours later (B) (n=3/group). C. The fold changes in *MMP2*, *MMP9*, and *TIMP2* expression levels in murine WR21 cells after 48 hours of treatment with KI at 100 μM were determined using qPCR. Target gene expression levels were normalized to *BETA-ACTIN* expression in the same samples, and fold changes were compared to expression levels in control samples (n=3/group). D and E. The fold changes in *P21*, *P27*, and *CYCLIN D1* expression levels in the human A253 cell line (D) and murine WR21 cell line (E) after 48 hours of treatment with KI at 100 μM were evaluated using qPCR. Target gene expression levels were normalized to *BETA-ACTIN* levels in the same samples, and fold changes were compared to expression levels in control samples (n=3/group). *$p < 0.05$, ***$p < 0.001$ were determined using a one-way ANOVA test (to assess the impact of two independent variables on a dependent variable) or Student`s t-test (to compare the performance of two groups under different conditions), with the mean and ± standard deviation (SD) depicted.

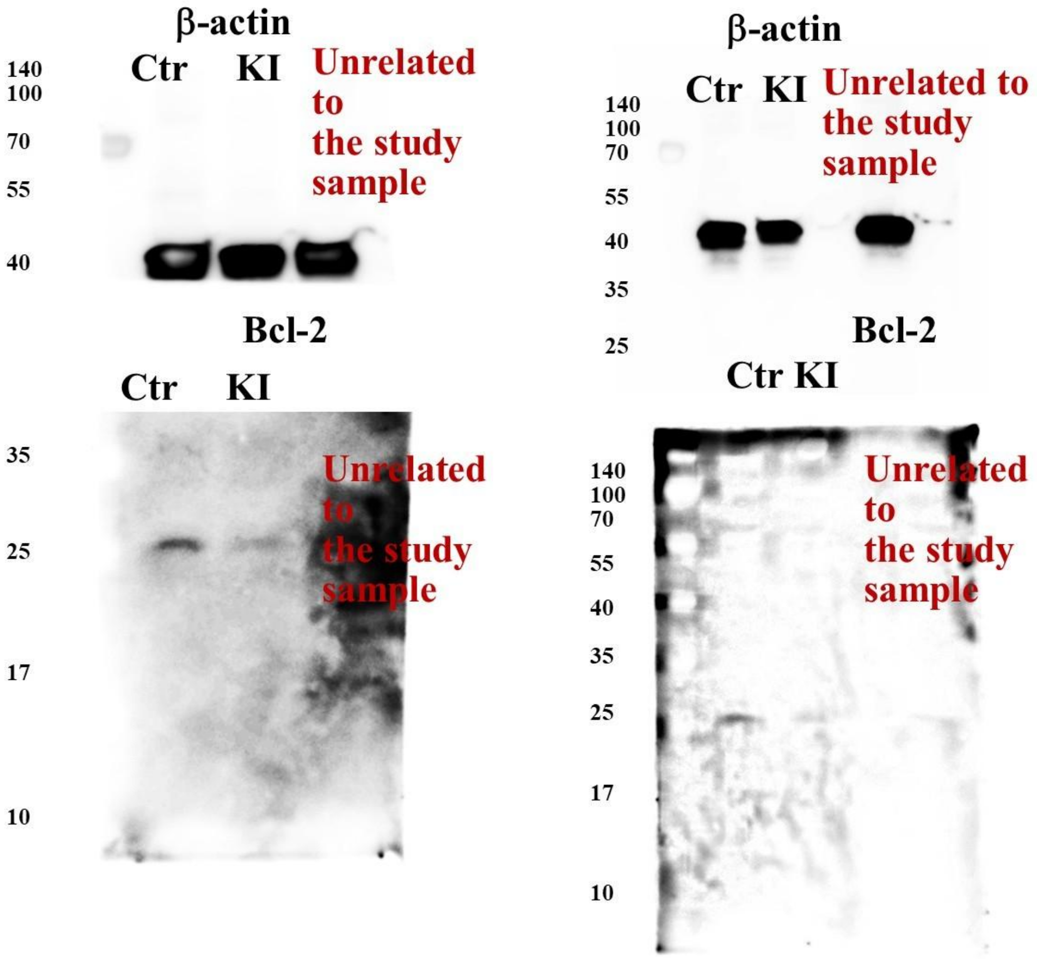

Supplementary Figure S2. Representative uncropped Western blots of Bcl-2 and β-actin in A253 cells treated with or without KI; results from two independent experiments are presented.

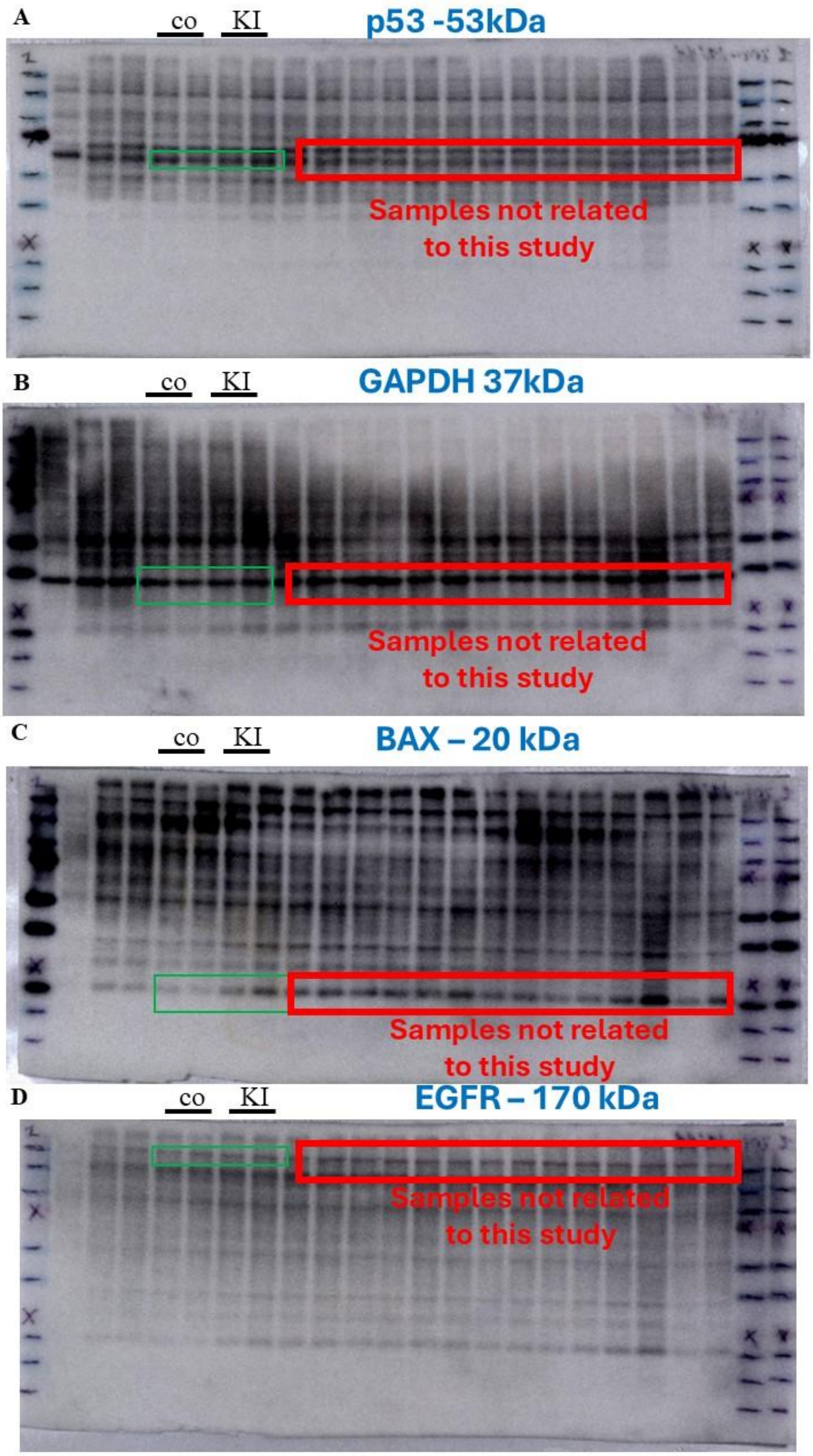

Supplementary Figure S3. Representative uncropped Western blot of p53 (**A**), GAPDH (**B**), BAX (**C**), and EGFR (**D**) of A253 cells treated with or without KI.

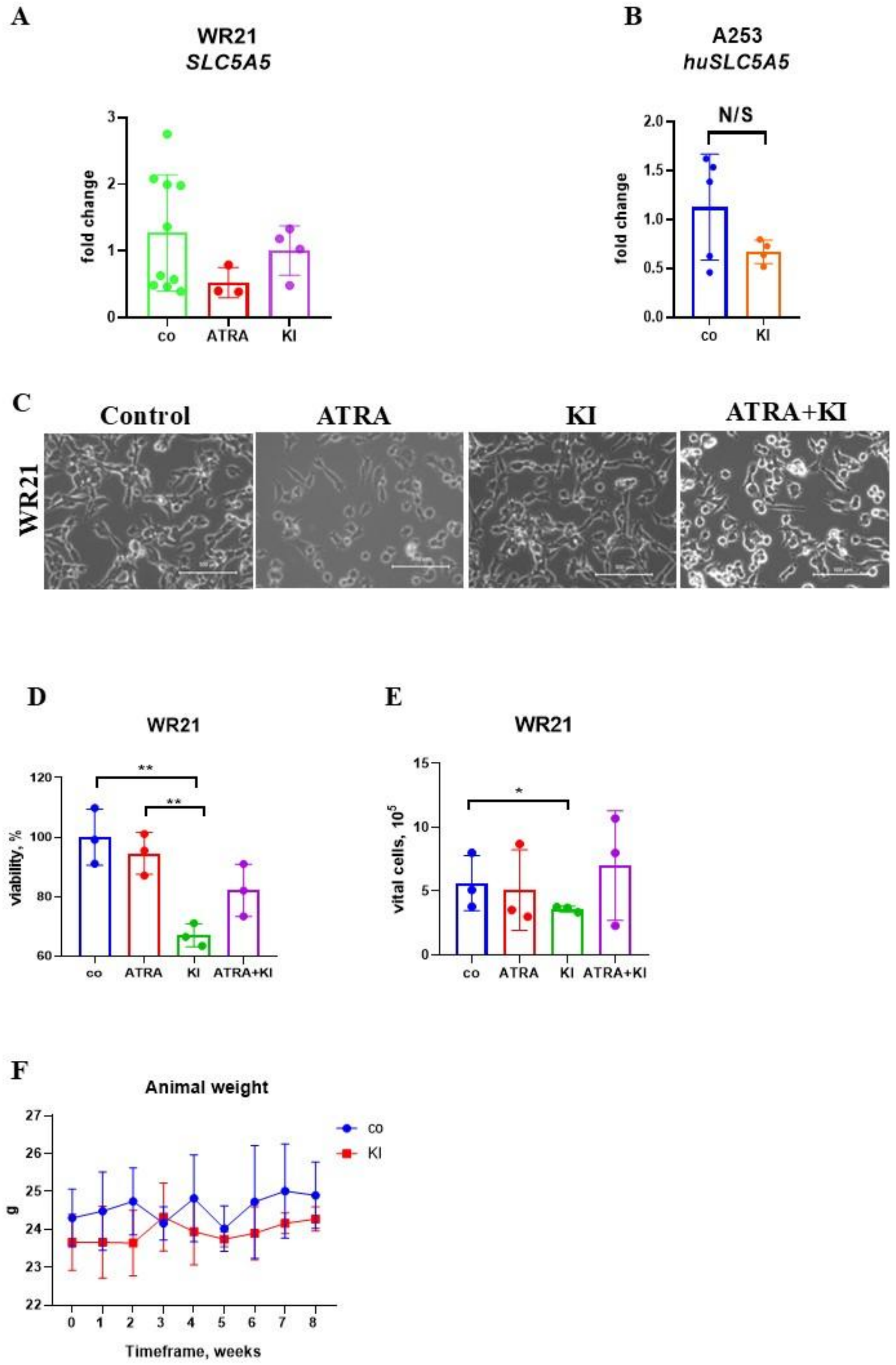

Supplementary Figure S4. No changes in *SLC5A5* expression or salivary gland cancer cell proliferation following ATRA or KI treatment. A and B. Fold changes in *SLC5A5* expression in control, KI-, and/or ATRA-treated WR21 (A) and A253 cells (B) were measured using qPCR (WR21, n= 10/3/4 per group; A253, n=5/4 per group). Target gene expression levels were normalized to *BETA-ACTIN* levels in the same samples, and fold changes were adjusted relative to the expression levels in control samples. (C) Representative light macroscopic images of murine WR21 cells 48 hours after incubation with KI (100 µM), ATRA (1 µM), their combination, and DMSO (ATRA control); scale bar = 100 µm. (D) The viability rate in WR21 cell cultures treated with ATRA and KI individually and in combination (n=3/group; viability in DMSO was set as 100% viability). (E) The total number of viable WR21 cells treated with the indicated drugs for 48 hours was assessed by trypan blue exclusion (n=3/group). (F) Weekly body weight monitoring control (co) and KI-treated (KI) mice receiving KI in drinking water throughout the experiment. The statistical significance is indicated as *p < 0.05, **p < 0.01, ***p < 0.001, ****p < 0.0001, based on a one-way ANOVA test (for assessing the effects of two independent variables on a dependent variable) or Student`s t-test (to compare the performance of two groups under different conditions), with mean ± SD depicted.

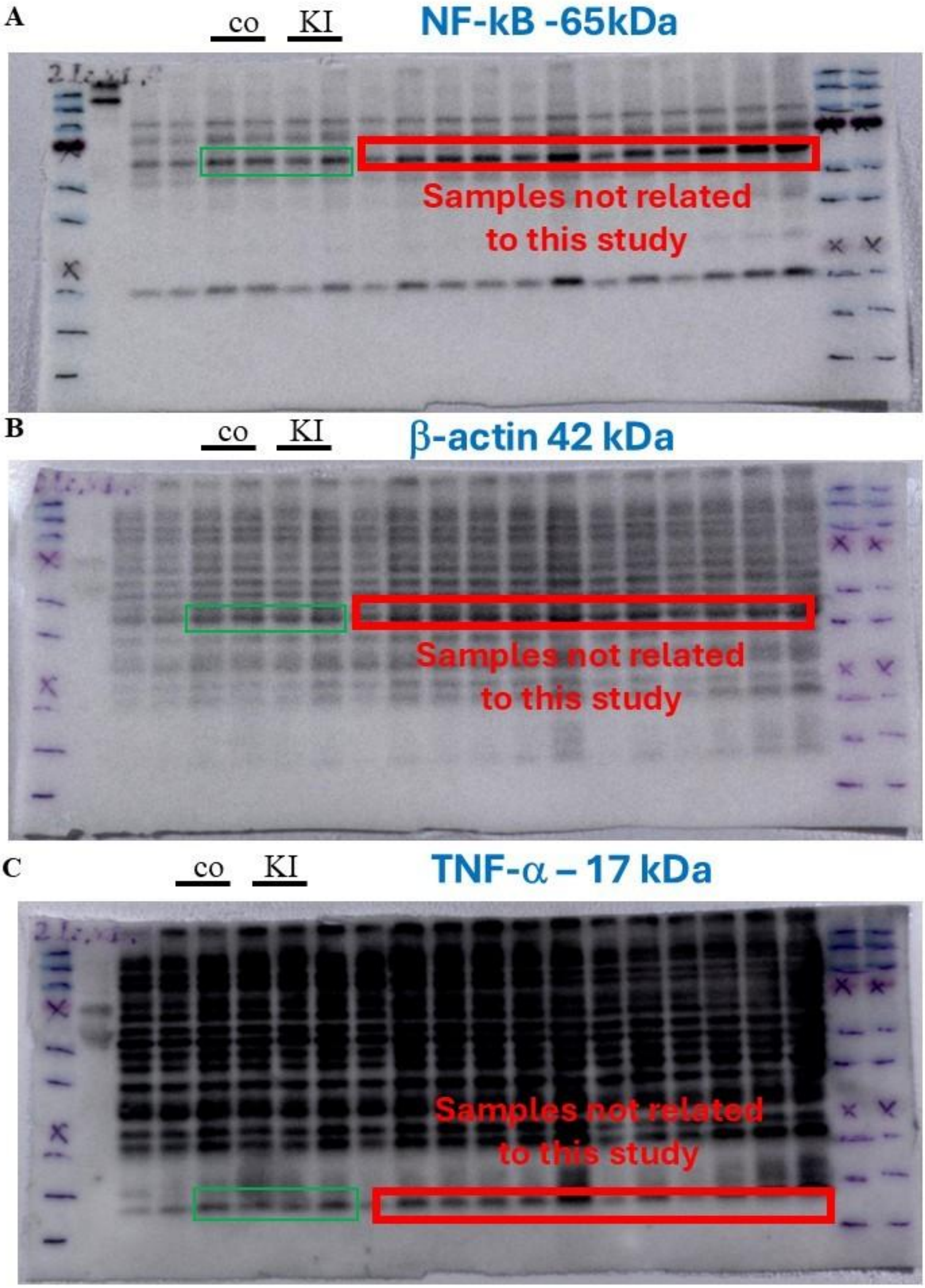

Supplementary Figure S5. Representative uncropped Western blot of NF-kB (**A**), β-actin (**B**), and TNF-α (**C**) of A253 cells treated with or without KI.

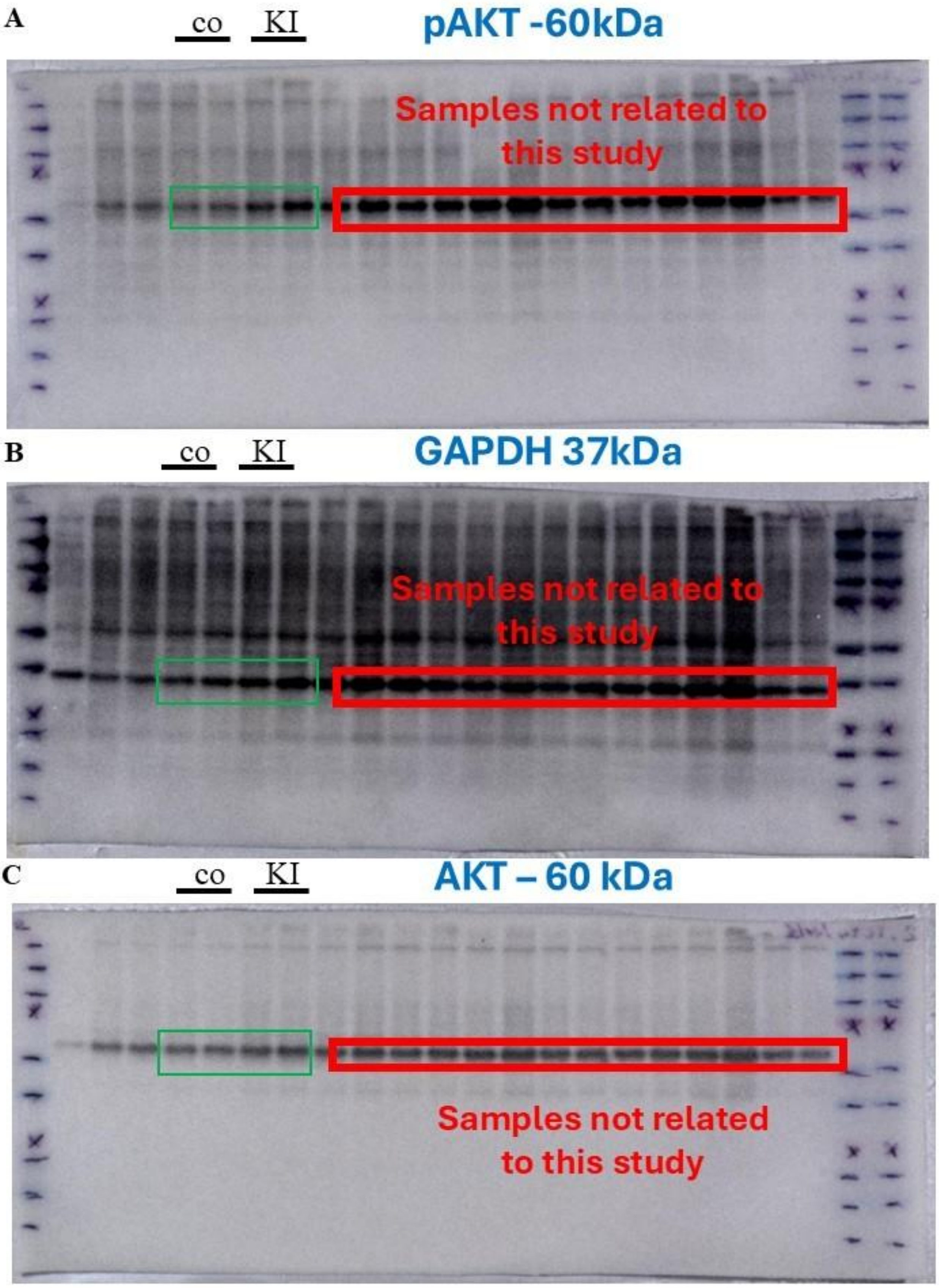

Supplementary Figure S6. Representative uncropped Western blot of pAKT (**A**), GAPDH (**B**), and AKT (**C**) of A253 cells treated with or without KI.
